# Differential impact of divalent metals on native elongating transcript sequencing (NET-seq) protocols for RNA polymerases I and II

**DOI:** 10.1371/journal.pone.0315595

**Published:** 2025-02-13

**Authors:** Abigail K. Huffines, David A. Schneider

**Affiliations:** Department of Biochemistry and Molecular Genetics, University of Alabama at Birmingham, Birmingham, AL, United States of America; Newcastle University, UNITED KINGDOM OF GREAT BRITAIN AND NORTHERN IRELAND

## Abstract

Throughout all domains of life, RNA polymerases (Pols) synthesize RNA from DNA templates, a process called transcription. During transcription, Pols require divalent metal cations for nucleotide addition and cleavage of the nascent RNA after misincorporation or polymerase stalling. Recently, several next-generation sequencing techniques have emerged to study transcription at single-nucleotide resolution *in vivo*. One such technique, native elongating transcript sequencing (NET-seq), allows for isolation of transcription elongation complexes associated with a specific Pol, defining polymerase occupancy on the DNA template. Originally developed to study RNA polymerase II (Pol II), NET-seq has been adapted for RNA polymerase I (Pol I) and bacterial RNA polymerase. We recently optimized Pol I NET-seq in *Saccharomyces cerevisiae*, however, we omitted nucleases and their metal cofactors, which are commonly used in Pol II NET-seq. Here, we investigated the effect of CaCl_2_ ± MNase and MnCl_2_ ± DNase I on Pol I occupancy. We found that exposure of Pol I to CaCl_2_ and MnCl_2_ during NET-seq caused a significant reduction in immunoprecipitation of nascent rRNA compared to the untreated control samples, with a more severe effect when incubated with MnCl_2_ vs. CaCl_2_. Surprisingly, in contrast to the Pol I results, we found that metal treatment during Pol II NET-seq did not have a significant effect on nascent transcript capture. Taken together, these observations reinforce the conclusion that transcription elongation complexes formed by Pols I and II have unique characteristics and emphasize the need to carefully consider experimental conditions deployed in all stages of nucleic acid library generation.

## Introduction

There are at least three eukaryotic nuclear RNA polymerases (Pols), RNA polymerase I (Pol I), RNA polymerase II (Pol II), and RNA polymerase III (Pol III), which synthesize unique RNA products. Pol I synthesizes the three largest ribosomal RNA (rRNA) species, Pol II synthesizes messenger RNA (mRNA) and many other regulatory RNAs, and Pol III synthesizes transfer RNA (tRNA), as well as the smallest rRNA. While transcription has been well-studied for many decades, high resolution techniques to investigate this process *in vivo* have only been used for the last ten years due to the rising popularity and affordability of next-generation sequencing (NGS).

Many techniques that measure Pol occupancy have recently emerged, including chromatin immunoprecipitation sequencing (chIP-seq), native elongating transcript sequencing (NET-seq), precision nuclear run-on sequencing (PRO-seq), and crosslinking and analysis of cDNA (CRAC). ChIP-seq is an adaptation of the classic chromatin immunoprecipitation (chIP) technique, in which crosslinking allows for the identification of regions in the DNA where a specific protein of interest binds [1–4]. ChIP-seq, which is the most commonly used of the listed techniques, identifies the occupancy of a large variety of proteins genome-wide *in vivo*. NET-seq was first established thirteen years ago to analyze Pol II occupancy on its target templates at single-nucleotide resolution via the isolation of transcription elongation complexes (ECs; a complex consisting of a transcribing Pol containing an RNA:DNA hybrid) and extraction of nascent RNAs in the absence of crosslinking [5]. In contrast, PRO-seq allows for the analysis of the occupancy of all Pols through the incorporation of a chain-terminating biotinylated nucleotide for isolation and identification of nascent RNAs [6]. Finally, CRAC relies on crosslinking to ensure the stability of the EC, followed by the immunoprecipitation (IP) of a specific Pol and nascent RNA isolation [7]. Beyond these examples, many other techniques continue to be developed to answer questions about how Pol occupancy changes under various conditions *in vivo*. Importantly, this new class of NGS technology yields novel insights into transcription properties *in vivo* that were previously inaccessible.

NET-seq was first established to probe for the occupancy of Pol II [5], but our lab recently adapted and optimized it to reproducibly determine Pol I occupancy at single-nucleotide resolution *in vivo* [8–12]. This technique takes advantage of the highly stable nature of the Pol I EC and therefore does not require any additional crosslinking. Instead, cells are cryogenically harvested and lysed, “freezing” Pol I on the rDNA template. Then, an IP is performed to isolate Pol I and the nascent RNA is extracted. Following this step, the protocol is very similar to other standard NGS library preparation methods, consisting of adapter ligation, reverse transcription, and library amplification and quality control. The 5’ end position of the output reads is complementary to the 3’ end of the nascent RNA, or the last incorporated nucleotide into the growing rRNA product. Therefore, these reads are mapped to the yeast genome to determine which single nucleotide position that the Pol I EC was occupying at the time of harvest.

Overall, NET-seq methods are very similar to the other techniques used to probe for Pol occupancy *in vivo* (chIP-seq, PRO-seq, and CRAC), with a few notable differences. One difference between NET-seq and chIP-seq or CRAC is the absence of crosslinking in NET-seq, which is used to ensure that the association between the protein of interest and either the DNA or RNA is not lost throughout the experiment. The ECs of Pols are highly stable [13], allowing for the IP of these intact complexes without crosslinking, therefore, this step has been deemed unnecessary in NET-seq experiments probing for Pol occupancy. One difference between NET-seq and PRO-seq is that in NET-seq, a single Pol of choice is isolated specifically, so modified nucleotides are not needed to distinguish between nascent and mature RNAs in the cell. In PRO-seq, either nuclei isolation or cell permeabilization is performed prior to adding these nucleotides [14]. While there is little research about the consequences of permeabilization to the cell, a recent study suggested that this process could cause cellular damage [15], therefore, while unlikely, it is unclear how this would affect gene regulation and transcription. Overall, each of these techniques possesses unique advantages and disadvantages that must be considered when designing an experiment.

The first NET-seq experiment was performed in *Saccharomyces cerevisiae* (yeast; [5]), which is the same model organism that our lab uses to determine Pol I occupancy. Yeast has long been considered to be an excellent model organism to study mechanisms of gene expression due to the high conservation of these processes between yeast and humans [16]. Since NET-seq was first developed, other labs have expanded this protocol to investigate Pol II occupancy in mammalian cells (mammalian NET-seq (mNET-seq); [17]). These variations, both in the organism used (yeast vs. mammalian cells) and Pol investigated (Pol I vs. Pol II), have resulted in substantial differences between NET-seq protocols. In the original published Pol II NET-seq protocol using yeast [5], cells were treated with deoxyribonuclease I (DNase I) and its required cofactor MnCl_2_. DNase I is a well-described endonuclease that nonspecifically cleaves accessible DNA. In contrast, mNET-seq publications have included a micrococcal nuclease (MNase) treatment with its required cofactor CaCl_2_ instead of DNase I + MnCl_2_ [18]. Similar to DNase I, MNase is an endonuclease, but has the ability to nonspecifically cleave accessible RNA and DNA. In our past NET-seq studies, we omitted these nucleases and divalent metal cation treatments. In both Pols I and II NET-seq protocols, there are several cryogenic steps (harvest and lysis) and for Pol I, EC incubation with EDTA (which sequesters divalent metal cations) to prevent the enzyme from adding nucleotides or cleaving the nascent RNA. It has long been established that *in vitro*, Pol I possesses robust intrinsic cleavage activity [19], where the presence of divalent metal cations, such as Mg^2+^, stimulates cleavage of the nascent rRNA when ECs are stalled or backtracked on the template. While developing our NET-seq methodology, we observed significantly enhanced reproducibility between replicates when EDTA was included. We hypothesize that the nascent RNA cleavage activity by Pol I is responsible for this effect, but to date, no one has evaluated the effects of divalent metal cations on NET-seq.

The goal of this study was to determine how exposure to various divalent metal cations and nuclease treatments affect NET-seq, since those treatments have been widely used in Pol II and bacterial RNA polymerase (RNAP) NET-seq protocols [20, 21]. Here, we tested the effects of CaCl_2_ ± MNase and MnCl_2_ ± DNase I treatments on Pol I NET-seq. We found that both CaCl_2_ and MnCl_2_ treatments caused a significant reduction in nascent rRNA recovery compared to the untreated control. The inclusion of nuclease treatments (MNase and DNase I) did not have an additional significant effect, but this could be due to the large loss of nascent RNA already established by the exposure to either CaCl_2_ or MnCl_2_. Furthermore, we found that MnCl_2_ caused a more severe effect on Pol I NET-seq compared to CaCl_2_ treatment, which could indicate that MnCl_2_ stimulates nascent RNA cleavage more robustly than CaCl_2_. Finally, in contrast to the observations for Pol I, treatment with either CaCl_2_ or MnCl_2_ during Pol II NET-seq did not cause a notable effect on nascent transcript capture as compared to the untreated control. Overall, these findings indicate that the exposure of Pol I to divalent metal cations during NET-seq library generation causes dramatic loss of nascent rRNA, reducing the reliability of the experiment. Furthermore, these results demonstrate that the transcription elongation complexes formed by Pols I and II have highly divergent biochemical properties. This observation using the *ex vivo* NET-seq methodology is consistent with previous findings *in vitro* [22].

## Results

### Pol I occupancy on the rDNA template is reproducibly mapped via NET-seq

The main goal of this study was to investigate the effect of common nuclease treatments and their divalent metal cation cofactors on Pol I occupancy in NET-seq experiments. In previous studies, we have shown that Pol I NET-seq is highly reproducible within strains or treatments, but an important first step is to confirm this before making comparisons between groups. Therefore, we first assessed the reproducibility of this experiment within treatment conditions. We compared four different treatments (CaCl_2_ ± MNase and MnCl_2_ ± DNase I) to an untreated control set of samples (five conditions total, each tested in triplicate).

**Fig 1:**
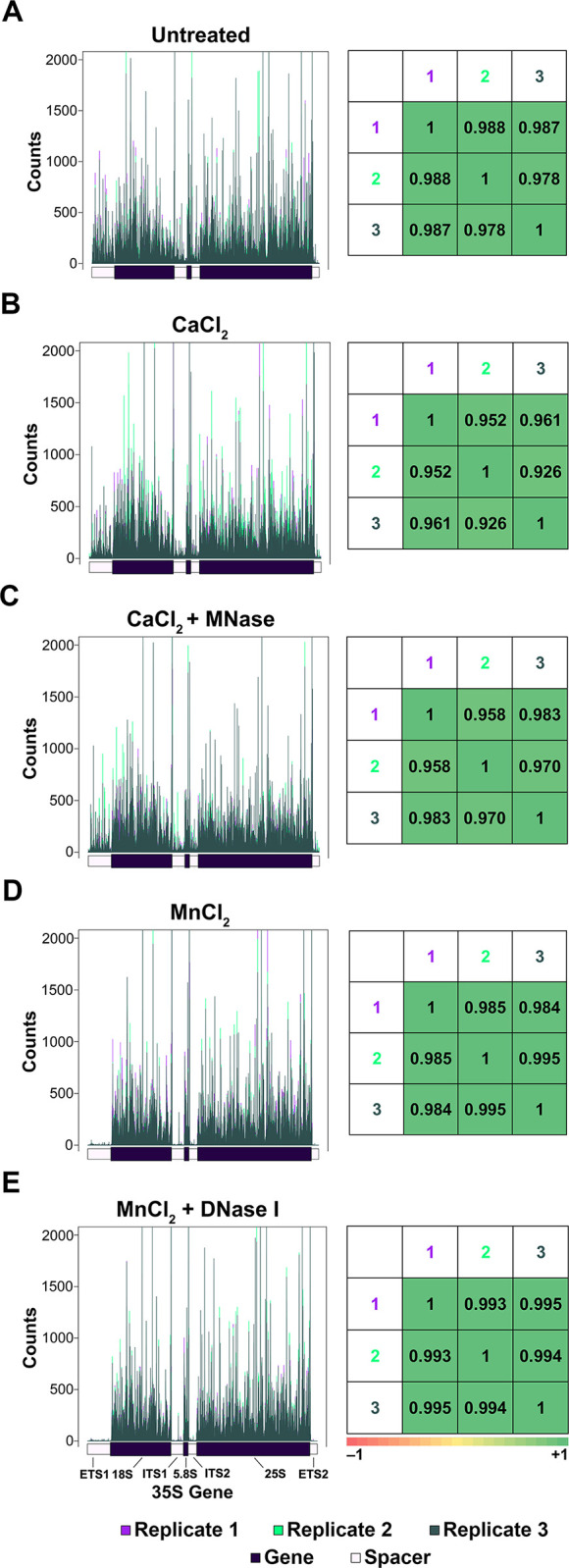
NET-seq is highly reproducible between biological replicates. For each treatment condition, overlaid occupancy patterns (left panels of A-E) are displayed for the three Pol I replicates. Spearman correlation coefficient values were calculated for the combination of all pairs within the biological replicates (right panels of A-E). Each panel (A-E) displays the data for a different treatment condition.

First, we normalized the number of reads at each position by the total number of reads for that sample and generated histograms to display the resultant counts (S1 Fig and left panels of Fig 1A–1E). These results are displayed as both individual (S1 Fig) and overlaid histograms (left panels of Fig 1A–1E) for an easy comparison between replicates. Overall, the histograms suggest that NET-seq is reproducible for samples within the same treatment group based on the similarity in occupancy patterns visible among triplicates. Next, we ran the Spearman correlation test to mathematically probe for the similarity between samples of the same treatment group. We determined that for all treatment groups, the Spearman correlation coefficient values were greater than 0.925 between samples, with over half of the values being greater than 0.980, confirming that NET-seq is highly reproducible among biological replicates (right panels of Fig 1A–1E). We also performed the Spearman correlation test for each of the three untreated samples vs. the treated samples (S2 Fig) and found that all resulting values were below 0.925, with the vast majority of them being less than 0.900. Collectively, these data indicate that NET-seq reproducibly probes for Pol I occupancy within the treatment groups tested in this study.

### Nascent RNA recovery is reduced after exposure to divalent metal cations

**Fig 2:**
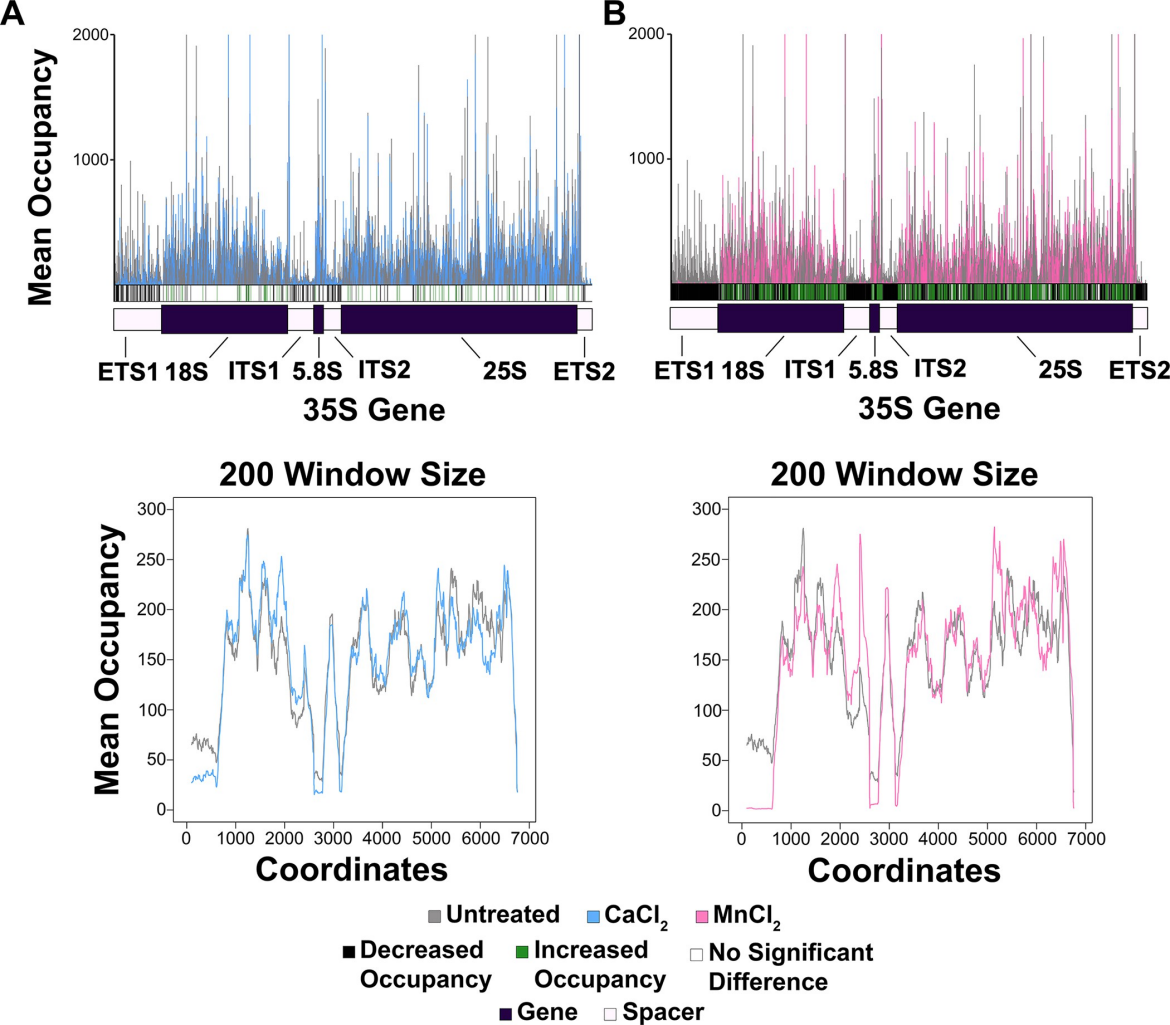
CaCl_2_ and MnCl_2_ treatment significantly reduced Pol I occupancy on the rDNA template. (A) Top panel: the mean Pol I occupancy was plotted for both the untreated and CaCl_2_ treated samples. At each position within the rDNA template, a t-test was performed, and a *p*-value was calculated. The *p-*value was adjusted via the p.adjust function in RStudio (method = “BH”). If the *p*-value < 0.05, that was deemed a significant difference between the two treatment groups and was indicated with either a green (increased occupancy) or black (decreased occupancy) line below the histogram for the CaCl_2_ treated samples with respect to the untreated samples. Bottom panel: the moving average across 200 nucleotides was plotted for both the untreated and CaCl_2_ treated samples. (B) Plots were generated as described in (A) for the untreated samples vs. MnCl_2_ treated samples.

After confirming that NET-seq was reproducible within treatment groups, we next investigated the effect of divalent metal cations (either CaCl_2_ or MnCl_2_) on Pol I occupancy. To do this, we compared the untreated samples to those treated with CaCl_2_ (Fig 2A and S3A–S3D Fig) or MnCl_2_ (Fig 2B and S3E–S3H Fig). It is important to note that the starting material for each experiment was the same, WT yeast cells grown to the same density and harvested under identical conditions. All variables examined in this study were present after lysis during library preparation (*ex vivo*).

First, the mean occupancy of the biological replicates was plotted for either the untreated or the CaCl_2_/MnCl_2_ treated samples, and these data were overlaid (top panels of Fig 2A and 2B). At each position, a t-test was performed to determine whether there was a significant difference in Pol I occupancy between the untreated and treated samples. We used the p.adjust function in RStudio (with the method = “BH”) to generate adjusted *p-*values. If significance was determined (*p*-value < 0.05) in the treated vs. untreated samples, this was indicated as either a black line (decreased occupancy), a green line (increased occupancy), or a white line (no significant change in occupancy) in the significance bar included below the histogram. When CaCl_2_ or MnCl_2_ were added to the experiment, there was a significant reduction in nascent RNA detected in the four spacer regions of the rDNA (external transcribed spacer 1 (ETS1), internal transcribed spacer 1 (ITS1), internal transcribed spacer 2 (ITS2), and external transcribed spacer 2 (ETS2)). These differences are reflected by the black lines in the significance bar. Interestingly, we also found that the treatment of MnCl_2_ (with almost solid black sections in the significance bar for the four spacer regions) seemed to cause a more drastic effect than CaCl_2_, which is discussed further below (Fig 5).

The spacer regions are especially important for the analysis of Pol I NET-seq since we have previously demonstrated that mature rRNA products cannot be excluded from samples during preparation and we are unable to distinguish between reads that originate from mature vs. nascent rRNA in our analysis [12]. It has been well-established that rRNA processing begins co-transcriptionally, and the four spacer regions are rapidly processed and removed prior to maturation of the rRNA. Therefore, we would expect that essentially all of the reads coming from mature rRNA contamination would map to the gene regions. Furthermore, we previously confirmed this prediction by performing NET-seq with a strain of yeast containing untagged Pol I [12]. We found that even when Pol I was not epitope-tagged, there was an enrichment of reads mapping to the gene regions of the rDNA and there were very few reads mapping to the spacer regions. Therefore, analysis of the spacer regions provides more confidence that the reads reflect Pol I occupancy, without potential artifacts from mature rRNA contamination.

Therefore, keeping our previous discovery that very little mature rRNA contamination maps to the spacer regions in mind, the simplest interpretation of these data (Fig 2 and S3 Fig) is that the decrease in NET-seq signal across all four spacer regions indicates that the exposure of Pol I to CaCl_2_ or MnCl_2_ causes eviction of Pol I from the rDNA. It is important to note that there is an apparent increase in reads mapping back to the gene regions in both the CaCl_2_ and MnCl_2_ treated samples vs. the untreated control samples, which is likely due to sequencing artifacts. The number of raw reads for each sample ranged from approximately 13–40 million, regardless of the treatment condition. While there is a significant decrease in the number of reads mapping to the spacer regions in the treated samples, the overall number of raw reads was relatively the same for these libraries as compared to the control samples. Therefore, to maintain a similar number of overall reads in the treated samples, it is likely that the majority of these reads have originated from the mature rRNA (which mostly align to the gene regions), resulting in the patterns observed in Fig 2. To complement this finding, we generated moving average plots across 200 nucleotides (Fig 2A and 2B, bottom panel). For these graphs, the average mean Pol I occupancy across the first 200 positions (1–201) of the rDNA for both the untreated and CaCl_2_/MnCl_2_ treated samples were plotted as the first data points. Next, the average mean Pol I occupancy across positions 2–202 were plotted for the untreated and treated conditions, and so on. These data confirm that when Pol I is exposed to CaCl_2_ or MnCl_2_, there is a substantial loss of signal compared to untreated samples, especially in the spacer regions.

### MNase and DNase I treatments did not have an observable effect on Pol I NET-seq

**Fig 3:**
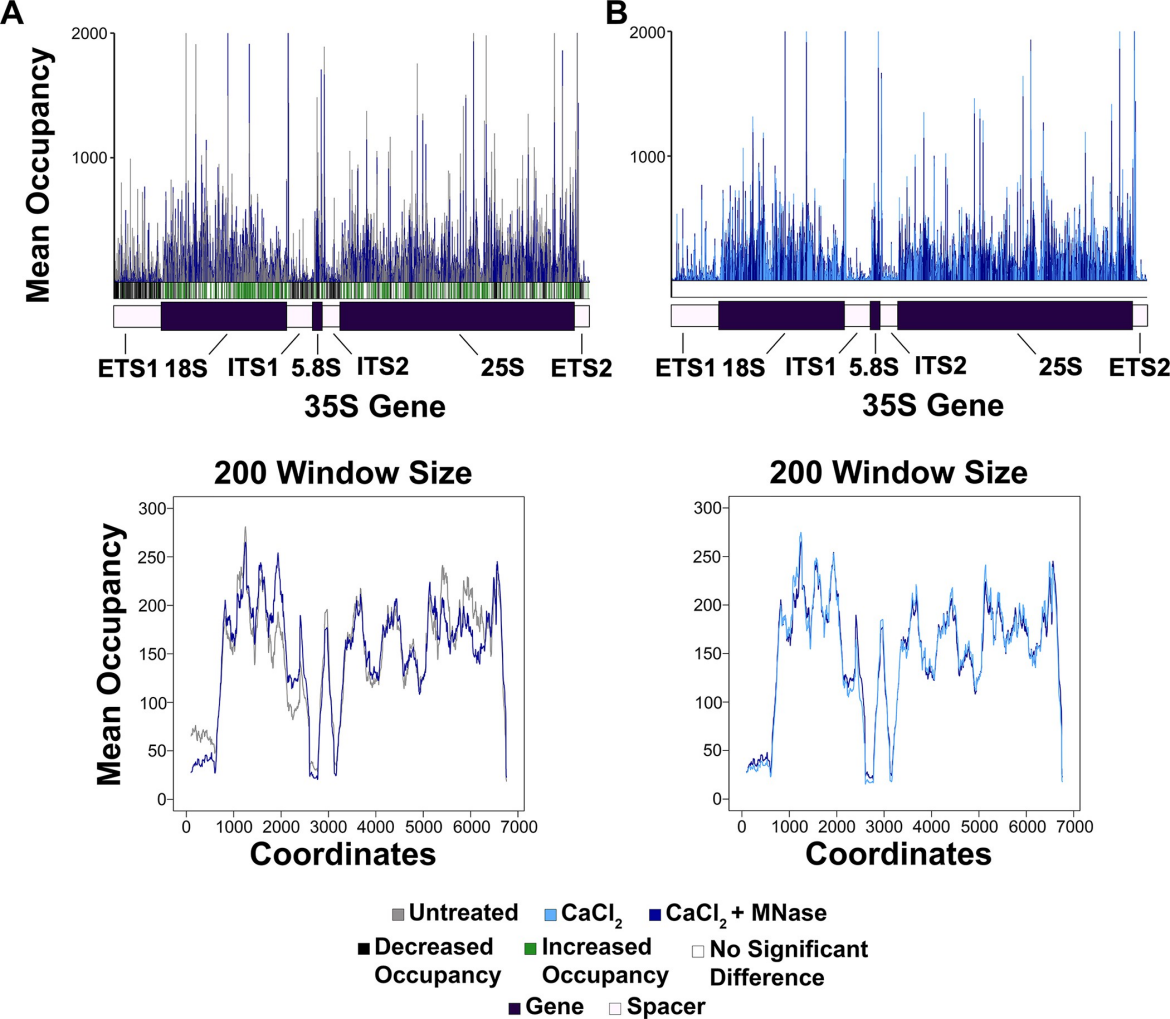
Pol I occupancy patterns were similar between CaCl_2_ and CaCl_2_ + MNase treatments. Mean comparison histograms and moving average plots for a 200-nucleotide window size were generated exactly as described in Fig 2 for (A) the untreated vs. CaCl_2_ + MNase treated samples and (B) CaCl_2_ vs. CaCl_2_ + MNase treated samples.

We next determined whether nuclease treatment affected Pol I NET-seq, as these enzymes are commonly used Pol II NET-seq. We plotted the NET-seq data as described above (mean comparison histogram and moving average plot) for the untreated samples vs. the CaCl_2_ + MNase treated samples (Fig 3A). There was a significant reduction in nascent RNA from the spacer regions of the CaCl_2_ + MNase treated samples as compared to the untreated samples (S4A–S4D Fig). However, when we compared the CaCl_2_ to the CaCl_2_ + MNase treated samples, the occupancy patterns were very similar between the two treatment groups (Fig 3B and S4E–S4H Fig). These data indicate that CaCl_2_ has a strong effect on Pol I NET-seq as demonstrated in Fig 2A and S3 Fig, but the addition of MNase had a negligible effect.

**Fig 4:**
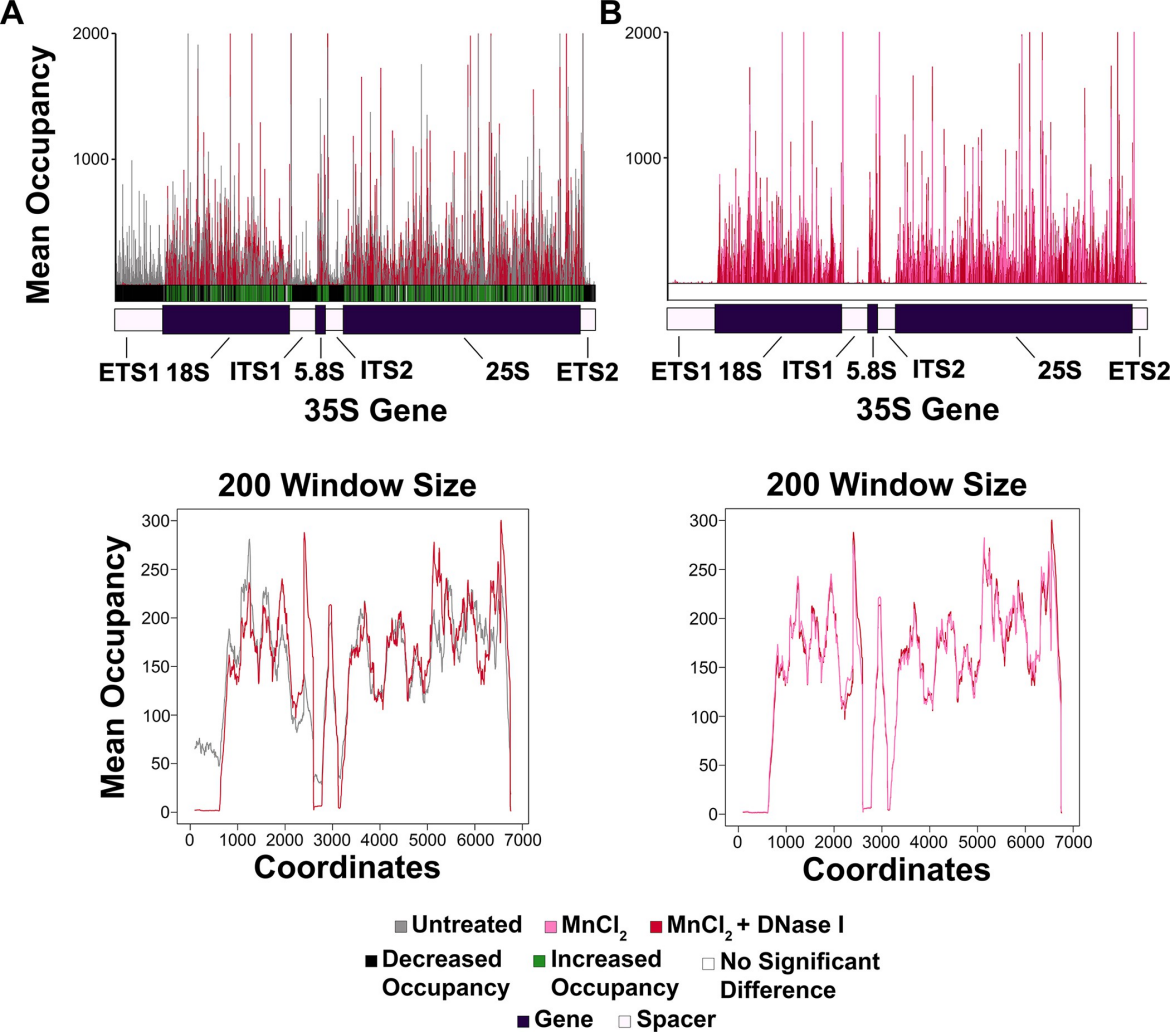
Pol I occupancy patterns were similar between MnCl_2_ and MnCl_2_ + DNase I treatments. Mean comparison histograms and moving average plots for a 200-nucleotide window size were generated exactly as described in Fig 2 for (A) the untreated vs. MnCl_2_ + DNase I treated samples and (B) MnCl_2_ vs. MnCl_2_ + DNase I treated samples.

We repeated this analysis for the MnCl_2_ + DNase I treated samples (Fig 4 and S5 Fig). Similarly, there was a significant reduction in Pol I NET-seq signal in the MnCl_2_ + DNase I as compared to the untreated control in the spacer regions (Fig 4A and S5A–S5D Fig). Therefore, the addition of DNase I did not cause substantial additional loss (Fig 4 and S5E–S5H Fig).

### MnCl_2_ treatment reduced Pol I occupancy more severely as compared to CaCl_2_

**Fig 5:**
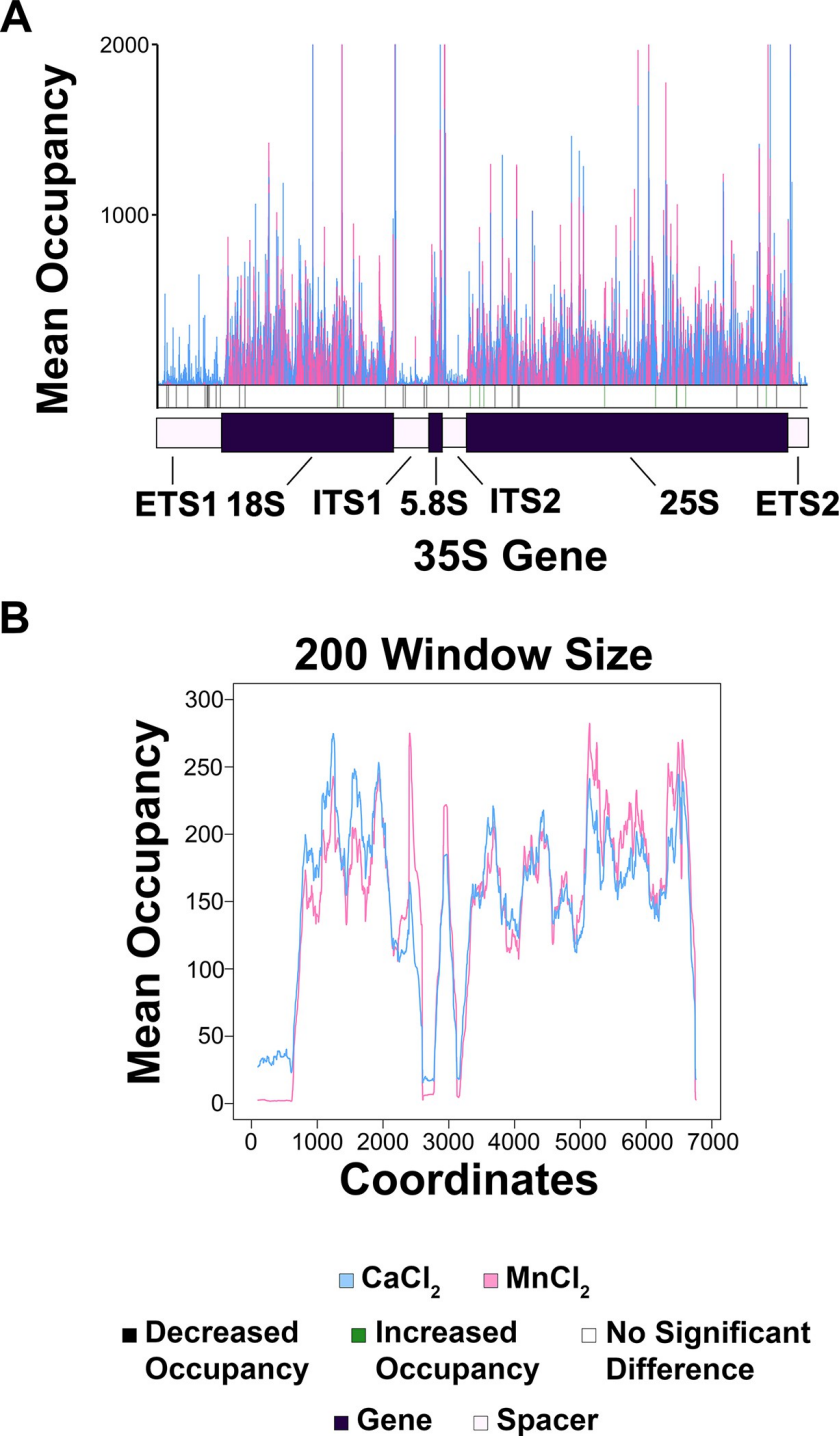
MnCl_2_ treatment causes a more severe reduction in Pol I occupancy as compared to CaCl_2_ treatment. (A) A mean comparison histogram was generated as described in Fig 2 to compare occupancy patterns between CaCl_2_ and MnCl_2_ treated samples. (B) A moving average plot with a 200-nucleotide window size was generated as described in Fig 2 to further assess differences in Pol I occupancy patterns between CaCl_2_ and MnCl_2_ treated samples.

To compare the effects of CaCl_2_ vs. MnCl_2_, we generated a mean comparison histogram and moving average plot as described above for the CaCl_2_ vs. MnCl_2_ treated samples (Fig 5 and S6 Fig). Though few positions were significantly different from each other between treatments, these data indicate that there was a greater reduction in recovery of nascent RNA in the spacer regions of MnCl_2_ vs. CaCl_2_ treated samples, especially evident in the patterns displayed in the moving average plot (Fig 5B).

To complement the findings from Figs 2–5, we performed a principal component analysis (PCA) to examine the clustering between all treatment groups (S7 Fig). The PCA plot demonstrated that the CaCl_2_ and CaCl_2_ + MNase samples clustered together, the MnCl_2_ and MnCl_2_ + DNase I clustered together, and all of these data sets were distinct from the untreated samples (S7 Fig). These data further support the conclusion that Pol I NET-seq is significantly impaired by exposure to divalent metal cations, and that the addition of either MNase or DNase I do not cause further significant changes to the data.

Taken together, these findings suggest that Pol I ECs cannot be reliably isolated in the presence of divalent metal cations. The simplest explanation for this observation is that Pol I displays robust nascent RNA cleavage activity. This cleavage activity requires divalent metal cations in the active site of the enzyme. When incubated with metals such as CaCl_2_ and MnCl_2_, this cleavage activity is activated and Pol I ECs ultimately degrade the nascent RNA. The observation that MnCl_2_ induces greater nascent RNA loss than CaCl_2_ suggest that Mn^2+^ may activate cleavage more robustly than Ca^2+^. This hypothesis is the subject of further investigations *in vitro*.

### In contrast to Pol I results, divalent metal cations do not have a significant effect on Pol II NET-seq experiments

The majority of published NET-seq studies are focused on Pol II and often include treatment with either CaCl_2_ or MnCl_2_. Despite the widespread use of these metals, their effect on Pol II NET-seq has not been tested. Considering our findings that the inclusion of either CaCl_2_ or MnCl_2_ causes a significant loss of rRNA recovery during Pol I NET-seq (Figs 2–5), it is critical to determine whether these metals alter Pol II results as well.

**Fig 6:**
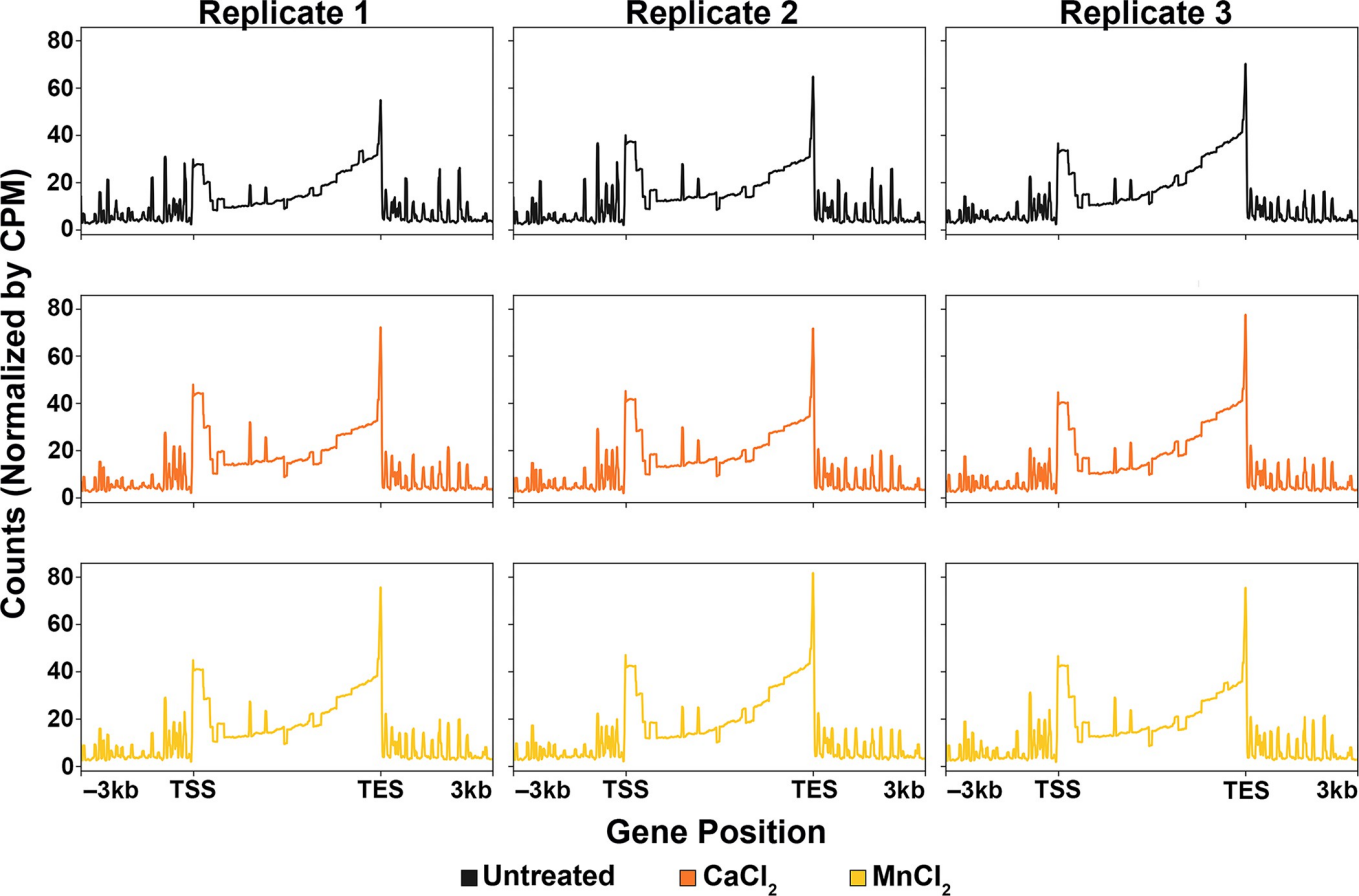
Pol II occupancy is not significantly altered by treatment with divalent metal cations. Pol II NET-seq was performed in triplicate for three different treatment conditions: untreated, CaCl_2_-treated, and MnCl_2_-treated. Counts were normalized by CPM and visualized by metagene analysis using the deepTools package via RStudio. Replicates are divided by column, while treatment conditions are divided by row. Occupancy patterns are included for 3 kilobases up- and downstream of the transcription start site (TSS) and the transcription end site (TES), respectively.

To test whether metal treatment effects were specific to Pol I experiments, we performed NET-seq using a strain of yeast carrying an HA-tag on the second largest subunit of Pol II, Rpb2. We prepared Pol II NET-seq libraries with three treatment conditions (untreated, CaCl_2_, and MnCl_2_) in triplicate and then used multiple tools from the deepTools package [23] to perform a metagene analysis for all samples. Interestingly, we found that Pol II occupancy patterns were essentially the same in CaCl_2_/MnCl_2_-treated vs. untreated samples (Fig 6). Surprisingly, this was the opposite of what we observed for Pol I experiments (Figs 2–5). To complement these findings, we used deepTools to perform a PCA (S8 Fig). The PCA plot demonstrates that the majority of the variation between all samples, regardless of treatment, was accounted for by PC1 (represented on the x-axis). All of the samples were plotted to nearly the same x-coordinate, therefore, all Pol II samples were highly similar. These contrasting results between Pols I and II further demonstrate that while similar in structure and function, these two enzymes have diverged from one another, a concept that has been explored previously *in vitro* [24–26].

## Discussion

In this study, we found that the exposure of Pol I to divalent metal cations drives significant changes to nascent rRNA recovery during NET-seq library preparation. Additionally, we demonstrated that MnCl_2_ causes a more robust inhibition of Pol I NET-seq compared to CaCl_2_. These data suggest that perhaps different divalent metal cations may stimulate RNA cleavage by Pol I to variable degrees. The inclusion of nuclease treatments (MNase and DNase I) did not have any effect on Pol I NET-seq beyond the observed signal reduction already induced by their metal cofactors. Finally, we determined that the treatment of either CaCl_2_ or MnCl_2_ did not have an effect on Pol II NET-seq results, illustrating a difference between Pols I and II. Altogether, these findings indicate that Pol I is exceptionally sensitive to the presence of various divalent metal cations, and care should be taken when including these reagents in experiments investigating Pol I occupancy.

Divalent metal cations such as Mg^2+^, Ca^2+^, and Mn^2+^ are required to stimulate the enzymatic activity of not only Pol I, but of all nucleic acid polymerases [27–29]. The active center of the polymerase coordinates two metal cations and these metals are required to catalyze both nucleotide addition as well as nascent RNA cleavage [30]. The mechanism for nucleotide addition is highly conserved for all DNA and RNA polymerases across every domain of life. While the basic requirement of divalent metal cations for polymerase activity has been well-established for many years and several *in vitro* studies have focused on the importance of these cations for DNA polymerases (reviewed in [27]), little is known about specific cation usage by RNA polymerases. Our findings in this study suggest a possible distinction between the role and activity of Mn^2+^ vs. Ca^2+^, at least for Pol I. We found that while the addition of either metal caused a significant inhibition of NET-seq, MnCl_2_ incubation almost completely evicted Pol I from the template, while CaCl_2_ reduced nascent RNA recovery to a lesser degree. One interpretation of this significant change is that Pol I cleavage activity is robustly activated by exposure to these divalent metal cations and exposure of Pol I to these metals may allow for degradation of these nascent transcripts by the polymerase. Additionally, these data suggest that MnCl_2_ may stimulate Pol I cleavage more robustly as compared to CaCl_2_. Our findings demonstrate that multiple divalent metal cations can be used to stimulate the activity of the polymerase, which has not been previously established for Pol I. Furthermore, these data are consistent with ongoing work from our lab investigating the effect of divalent metal cations on Pol I activity. Altogether, these findings suggest that divalent metal cations may have different effects on the enzymatic activity of Pol I and may allow for varied RNA cleavage rates.

Our observation that the inclusion of either CaCl_2_ or MnCl_2_ in Pol II experiments had little effect on occupancy is subject to several possible interpretations. First, while the role and importance of divalent metal cations during nucleotide addition is conserved across polymerases, the preference and requirement for certain metals is unknown and could be different for the three eukaryotic Pols. Second, we observed a drastic reduction in rRNA capture after metal treatment for Pol I, which we attribute to the robust intrinsic cleavage activity of the enzyme. Interestingly, Pol II is also capable of cleavage, but it accomplishes this through a transcription factor, TFIIS, which does not remain associated with Pol II at all times during transcription [31–33]. Perhaps the requirement of TFIIS recruitment by Pol II can explain the significant difference in NET-seq results observed here. Finally, previous work showed that Pol II forms transcription elongation complexes that are far more stable than those of Pol I *in vitro* [22]. It is possible that these intrinsic differences in the enzymes relate to the observed divalent metal sensitivity observed in NET-seq. Furthermore, to date, there have been no published studies on NET-seq probing for the occupancy of Pol III, which possesses intrinsic cleavage properties similar to Pol I through its C11 subunit [34]. This could be an avenue of future interest, especially for comparative studies between the three Pols. Overall, our findings represent the importance of studying these enzymes individually, as they have diverged to play distinct roles in the cell.

Here we investigated the effect of two commonly used nucleases from other NET-seq protocols, MNase and DNase I. These nucleases both possess endonuclease activity and cleave either DNA only (DNase I) or both DNA and RNA (MNase) non-specifically. We found that the use of nucleases in our experiment was detrimental due to the required addition of their divalent metal cation cofactors. From these data, it is difficult to determine whether the addition of these nucleases could provide a beneficial role because of the significant reduction in Pol I occupancy on the rDNA template induced by the exposure to either MnCl_2_ or CaCl_2_ (Figs 2–4 and S3–S6 Figs).

One of the reasons that these nucleases have previously been included in Pol II NET-seq studies is to eliminate potentially contaminating DNAs from the sample prior to library generation. If there is DNA carryover, this could pose an issue in our Pol I experiments as well. The goal of NET-seq is to map the last incorporated nucleotide of the RNA transcript, so contaminating DNAs could artificially alter these results if they are present in the sample without removal. In our data sets, the vast majority of reads map to one strand of the DNA (the + strand) rather than both strands. This observation suggests that it is unlikely there is much, if any, DNA contamination present in our samples even without a nuclease treatment step. Therefore, at least for Pol I, the addition of nucleases in the NET-seq protocol is unnecessary and, as this study reveals, detrimental.

Altogether, the findings in this study provide new insight into the use of divalent metal cations and nucleases in NET-seq experiments used to probe for Pols I and II occupancy. While we only compared the effects of these metals on Pols I and II in this study, our results may apply to other enzymes across the three domains of life, and further testing is necessary prior to performing NET-seq with metals for different polymerases. In addition, these findings could be expanded to other techniques that aim to investigate Pol occupancy, such as chIP-seq, PRO-seq, and CRAC. Collectively, these data suggest that the exposure of Pol I to divalent metal cations causes a significant loss of nascent RNA recovery, and caution should be exercised when using nuclease treatments in NGS techniques.

## Materials and methods

### Cell growth and harvest

NET-seq was performed exactly as previously published [8–12], with a detailed protocol and buffer recipes included in the Supporting Information (S1 Text and S1–S14 Tables). The protocol described in this peer-reviewed article is also published on *protocols*.*io* (DOI: dx.doi.org/10.17504/protocols.io.bp2l6dd91vqe/v1). In brief, *Saccharomyces cerevisiae* cells (Pol I NET-seq: (His)7-(HA)3-RPA135::TRP1); Pol II NET-seq: RPB2-(HA)_3_-(His)_7_::HIS3Mx6) were grown to A_600_ = 0.3 at 30°C with rotation. Once cells reached the desired optical density, they were quickly harvested via filtration, flash frozen, and stored at -80°C until lysis.

### Cell lysis

Cells were lysed under cryogenic conditions either using a Mikro-Dismembranator II (for Pol I NET-seq) or a CoolTeenPrep adapter (for Pol II NET-seq). For the Mikro-Dismembranator II, the machine was set on maximum speed (16 mm) and ten lyse/rest cycles (one minute of lysis, one minute submerged in liquid nitrogen) were conducted. For the CoolTeenPrep adapter, five lyse/rest cycles (one minute of lysis, one minute of tubes resting on dry ice) were performed. After lysis was completed, grindate weight was recorded and cells were stored at -80°C.

### Blocking beads for immunoprecipitation

One day prior to immunoprecipitation and RNA extraction, Pierce Anti-HA magnetic beads were blocked overnight with BSA. For blocking, lysis and blocking buffers (as specified in S2 and S3 Tables) were prepared. The appropriate amount of magnetic beads for each sample ((sample grindate weight * 5) * (0.04)) was transferred to a 1.5 mL microcentrifuge tube (one tube per sample) and washed three times with 1 mL of lysis buffer for two minutes at 4°C with inversion on a tube rotator. Finally, lysis buffer was removed, 0.5 mL of blocking buffer was mixed with the beads in each tube, and beads were blocked overnight at 4°C with inversion on a tube rotator.

### Immunoprecipitation and RNA extraction

Lysis buffers were prepared according to the recipes provided in the supporting information (S2 Table (with EDTA) and S4 Table (without EDTA)). Grindates were resuspended in the appropriate amount of ice-cold lysis buffer (5 mL * grindate weight) and were placed on ice. Methods for the CaCl_2_/CaCl_2_ + MNase treated samples were adapted from a previous publication where mNET-seq was performed [18]. For those samples, a final concentration of 5 mM CaCl_2_ was added and a final concentration of 100 U/mL MNase was added to the CaCl_2_ + MNase samples only. Both the CaCl_2_/CaCl_2_ + MNase treated samples were incubated at 37°C for 2 minutes, a final concentration of 5 mM EDTA (pH 8.5) was added to all samples, and they were placed on ice. For the MnCl_2_/MnCl_2_ + DNase I samples, methods were adapted from the original protocol published to study Pol II occupancy in yeast [5]. For these samples, a final concentration of 10 mM of MnCl_2_ was added and a final concentration of 100 U/mL DNase I was added to the MnCl_2_ + DNase I samples only. The MnCl_2_/MnCl_2_ + DNase I samples were incubated for 20 minutes on ice. A final concentration of 5 mM EDTA (pH 8.5) was added to the samples, and they were incubated for an additional 30 minutes on ice. All samples (both untreated and treated) were then centrifuged at 16,873 x g for 15 minutes at 4°C. The supernatant for each sample was transferred into a 15 mL conical tube with the pre-blocked beads. Samples and beads were incubated for 3 hours at 4°C with inversion on a tube rotator.

After 3 hours elapsed, beads were washed 4 times with 1X wash buffer (S5 Table). After the final wash, beads were resuspended in 500 μL TES. RNA was extracted using an acidic phenol (pH 4.3)/chloroform extraction (2 total phenol and 2 total chloroform extractions). After the final chloroform extraction, the aqueous layer for each sample was transferred to a new tube. To each sample, 10 μL of a glycoblue solution (a 1:5 dilution with sterile MilliQ water) and 1.4 mL of an ammonium acetate precipitation solution was added. Samples were precipitated at -80°C overnight or longer (samples can be precipitated for months under these conditions).

### Linker ligation, enzymatic linker digestion, and zinc fragmentation

Samples were spun at 16,873 x g for 1 hour at 4°C. RNA pellets were washed a total of two times with 75% ethanol. Once the ethanol had evaporated after the final wash, pellets were resuspended in 10 mM Tris-HCl (pH 6.9), and the RNA concentration and quality was determined using a nanodrop. The ligation mix was prepared according to the recipe provided in the supporting information (S6 Table) without the addition of the UMI linker (see oligo sequence in S12 Table). After denaturing the UMI linker at 80°C for 3 minutes, it was added to the ligation mix. RNA samples were denatured at 80°C for 2 minutes, and then mixed with 9 μL of the ligation mix and 1 μL of T4 RNA Ligase 2, truncated. Finally, samples were incubated at 25°C for 3 hours for ligation of the UMI linker to the RNA products.

After the ligation was complete, several steps were performed to enzymatically digest the excess linker in the samples. First, 2 μL of 5’ Deadenylase was added to each sample and samples were incubated at 30°C for 45 minutes. Next, samples were diluted 2.5X with sterile MilliQ water (13.2 μL) that was supplemented 0.6X with NEBuffer 2 (19.8 μL). To each sample, 2 μL of RecJ_f_ was added and then samples were incubated at 37°C for 45 minutes. Finally, 2.2 μL of fragmentation solution was added to each sample and samples were incubated at 70°C for 16 minutes. After fragmentation, samples were immediately placed on ice, and 2.5 μL of 200 mM EDTA (pH 8.5) was added to each sample to quench the reaction. Samples were transferred to a new tube with 1 μL of glycoblue and 360 μL of ammonium acetate precipitation solution. Samples were precipitated at -80°C overnight or longer (samples can be precipitated for weeks or months under these conditions).

### Reverse transcription and size selection

Samples were centrifuged and washed two times with 75% ethanol the same way as described in the “linker ligation, enzymatic linker digestion, and zinc fragmentation” section. After all of the residual ethanol evaporated from sample tubes, pellets were resuspended in 10 μL of 10 mM Tris-HCl (pH 6.9). The reverse transcription and RNasin/DTT mixes were prepared based on the recipes provided in the supporting information (S7 and S8 Tables). Each sample was combined with 5.4 μL of the reverse transcription mix, and samples were incubated at 65°C for 5 minutes. To each sample, 1.32 μL of the RNasin/DTT mix and 0.82 μL of Superscript III Reverse Transcriptase were added. Samples were incubated at 45°C for 30 minutes, and then 1.8 μL of 1 M NaOH was added to each tube. Finally, samples were incubated at 98°C for 20 minutes to complete the reverse transcription.

Following reverse transcription, samples were resolved on a 10% polyacrylamide gel. The gel was stained with SYBR gold, and a gel extraction was performed to excise samples between 120 bp and 600 bp. Gel slices were pulverized and combined with 500 μL of sterile MilliQ water. Gel slurries were first incubated at -80°C for 15 minutes, then at 70°C for 15 minutes, and finally at 30°C with rotation overnight. The next day, gel slurries were spun through a Costar Spin-X centrifuge tube filter to separate gel pieces from the liquid sample. Gel pieces were discarded, while liquid samples were combined with 32 μL of 3 M NaCl, 940 μL of 100% isopropanol, and 1 μL of glycoblue. Samples were precipitated at -80°C overnight or longer (samples can be precipitated for weeks or months under these conditions).

### Circularization and library amplification/preparation for sequencing

Samples were centrifuged and pellets were washed as described in the “linker ligation, enzymatic linker digestion, and zinc fragmentation” section. Pellets were resuspended in 15 μL of 10 mM Tris-OAc (pH 7.9). The circularization mix was prepared as described in the supporting information (S9 Table). Each sample was mixed with 4 μL of circularization mix and 1 μL of CircLigase ssDNA Ligase. Samples were incubated at 60°C for 1 hour, an additional 1 μL of CircLigase ssDNA Ligase was added to each sample, and then samples were incubated for an additional hour at 60°C. To inactivate the CircLigase ssDNA Ligase enzyme, samples were incubated at 80°C for 10 minutes.

Following circularization, the phusion master mix (S10 Table) and library amplification mixes were prepared as specified in the recipes included in the supporting information. For each sample, 1 μL of circularized DNA was mixed with 16.7 μL of a unique library amplification mix prepared specifically for that sample. The libraries were amplified for a total of 25 cycles based on the protocol included in S11 Table. Finally, libraries were prepared for sequencing by using PCRClean DX Beads according to the manufacturer’s specifications.

### NET-seq data analysis

Libraries were sequenced as previously described using the Illumina NovaSeq 6000 [35].

Initial data analysis was performed exactly as previously published with the same package options [10–12] for both Pols I and II data. In brief, sequencing read quality was first investigated by generating quality reports for each sample using FastQC (version 0.11.7-Java-1.8.0_74 [36]). Next, reads were deduplicated based on the UMI sequence with fqtrim (version 0.9.7 [37]). The 5’ and the 3’ adapters were removed from remaining reads by using cutadapt (version 3.4 [38]). Reads were then aligned to the yeast genome (*Saccharomyces cerevisiae* genome assembly 64-1-1) with STAR (version 2.7.1a-foss-2018b [39]).

For Pol I NET-seq, files were sorted and indexed with SAMtools (version 1.6-intel-2016a [40]) and BAM files were converted to BED files with BEDTools (version 2.28.0 [41]). Genome occupancy coverage files were generated for the positive and negative strands for each sample using BEDTools. Visualization of occupancy patterns was performed using RStudio (version 1.3.959) and R (4.0.2). Plots were generated as previously described [10–12] and packages and versions used are included in S14 Table. Raw FASTQ files can be accessed through NCBI’s Gene Expression Omnibus via series accession number GSE216460.

For Pol II NET-seq, SAMtools (version 1.6-intel-2016a [40]) was used to index aligned BAM files. Next, a series of tools from the deepTools package [23] were used to conduct and visualize the metagene analysis: bamCoverage (with parameters “—Offset 1—normalizeUsing CPM”), the scale-regions subtool within the computeMatrix tool (with parameters “-b 3000 -a 3000—regionBodyLength 5000—skipZeros”), and plotProfile (with parameters “—numPlotsPerRow 3”). Samples were normalized by CPM during the bamCoverage step. The multiBigwigSummary and plotPCA tools from deepTools were used to perform the PCA. Raw FASTQ and processed bigWig files are available through NCBI’s Gene Expression Omnibus via series accession number GSE267082.

## Supporting information

S1 Table 10X lysis buffer. (PDF)

S2 Table 1X lysis buffer with EDTA included. (PDF)

S3 Table 1X blocking buffer. (PDF)

S4 Table 1X lysis buffer without EDTA. (PDF)

S5 Table 1X wash buffer. (PDF)

S6 Table Ligation mix for a single sample. (PDF)

S7 Table Reverse transcription mix for a single sample. (PDF)

S8 Table RNasin/DTT mix for a single sample. (PDF)

S9 Table Circularization mix for a single sample. (PDF)

S10 Table Phusion master mix for a single sample. (PDF)

S11 Table Library amplification cycle protocol. (PDF)

S12 Table Oligo sequences for the UMI linker and reverse transcription primer. (PDF)

S13 Table Oligo sequences for library amplification indices for each sample. (PDF)

S14 Table R packages and versions used in data analysis. (PDF)

S1 Fig Histograms for individual replicates display reproducibility within treatment conditions. (TIF)

S2 Fig Spearman correlation coefficient values for untreated vs. treated samples. (TIF)

S3 Fig Divalent cation treatment reduces Pol I occupancy across the spacer regions of the rDNA. (TIF)

S4 Fig CaCl_2_ treatment causes a significant reduction in Pol I occupancy, but the addition of MNase only results in mild changes to occupancy in the spacer regions. (TIF)

S5 Fig MnCl_2_ treatment causes a significant reduction in Pol I occupancy and DNase I does not have an additional effect in the spacer regions. (TIF)

S6 Fig MnCl_2_ treatment causes a more severe reduction in Pol I occupancy as compared to CaCl_2_ treatment in the spacer regions. (TIF)

S7 Fig Principal component analysis (PCA) plot for Pol I samples. (TIF)

S8 Fig PCA plot for Pol II samples. (TIF)

S1 Text (DOCX)
