# Supplementary material for: Differential impact of divalent metals on native elongating transcript sequencing (NET-seq) protocols for RNA polymerases I and II
Source: PLoS One. 2025 Feb 13;20(2):e0315595. doi: 10.1371/journal.pone.0315595 (PMC11824990; doi:10.1371/journal.pone.0315595)
Supplement: S1 Table — (PDF) [file pone.0315595.s001.pdf]

	**Final Concentration**
**Tris-HCl, pH 7.9**	200 mM
**Triton X-100 (Sigma, #T8787-100ML)**	4%
**NP-40 (Sigma, #NP40S-100ML)**	1%
**Sterile MilliQ Water**	up to volume
