# Supplementary material for: Differential impact of divalent metals on native elongating transcript sequencing (NET-seq) protocols for RNA polymerases I and II
Source: PLoS One. 2025 Feb 13;20(2):e0315595. doi: 10.1371/journal.pone.0315595 (PMC11824990; doi:10.1371/journal.pone.0315595)
Supplement: S3 Table — (PDF) [file pone.0315595.s003.pdf]

	**Final Concentration**
**10X Lysis Buffer Stock (Table S1)**	10%
**NH_4Cl**	100 mM
**Halt Protease Inhibitor (ThermoScientific, #78430)**	1%
**EDTA, pH 8.5**	5 mM
**RiboLock RNase Inhibitor (ThermoFisher, #EO0382)**	25 U/mL
**BSA**	10 mg/mL
**Sterile MilliQ Water**	up to volume
