# Supplementary material for: Differential impact of divalent metals on native elongating transcript sequencing (NET-seq) protocols for RNA polymerases I and II
Source: PLoS One. 2025 Feb 13;20(2):e0315595. doi: 10.1371/journal.pone.0315595 (PMC11824990; doi:10.1371/journal.pone.0315595)
Supplement: S6 Table — (PDF) [file pone.0315595.s006.pdf]

	**1X**
**50% PEG, MW 8000 (NEB, #B1004S)**	4.8 μL
**10X T4 RNA Ligase Buffer (NEB, #B0216S)**	2 μL
**20 μM UMI Linker**	1 μL
**RiboLock RNase Inhibitor (ThermoFisher, #EO0382)**	0.5 μL
**Sterile MilliQ Water**	0.7 μL
**Total Volume**	**9 μL**
