# Supplementary material for: Differential impact of divalent metals on native elongating transcript sequencing (NET-seq) protocols for RNA polymerases I and II
Source: PLoS One. 2025 Feb 13;20(2):e0315595. doi: 10.1371/journal.pone.0315595 (PMC11824990; doi:10.1371/journal.pone.0315595)
Supplement: S7 Table — (PDF) [file pone.0315595.s007.pdf]

	**1X**
**5X First Strand Buffer (Included in Superscript III Kit)**	3.28 μL
**10 mM each dNTPs (ThermoFisher, #R0181)**	1.64 μL
**10 μM NET2 Primer**	0.5 μL
**Total Volume**	**5.42 μL**
