# Supplementary material for: Differential impact of divalent metals on native elongating transcript sequencing (NET-seq) protocols for RNA polymerases I and II
Source: PLoS One. 2025 Feb 13;20(2):e0315595. doi: 10.1371/journal.pone.0315595 (PMC11824990; doi:10.1371/journal.pone.0315595)
Supplement: S8 Table — (PDF) [file pone.0315595.s008.pdf]

	**1X**
**RiboLock RNase Inhibitor (ThermoFisher, #EO0382)**	0.25 μL
**100 mM DTT (Included in Superscript III Kit)**	0.82 μL
**Sterile MilliQ Water**	0.25 μL
**Total Volume**	**1.32 μL**
