# Supplementary material for: Differential impact of divalent metals on native elongating transcript sequencing (NET-seq) protocols for RNA polymerases I and II
Source: PLoS One. 2025 Feb 13;20(2):e0315595. doi: 10.1371/journal.pone.0315595 (PMC11824990; doi:10.1371/journal.pone.0315595)
Supplement: S9 Table — (PDF) [file pone.0315595.s009.pdf]

	**1X**
**10X Circularization Buffer (Included in CircLigase Kit)**	2 μL
**1 mM ATP (Included in CircLigase Kit)**	1 μL
**500 mM $MnCl_2$ (Included in CircLigase Kit)**	1 μL
**Total Volume**	**4 μL**
