# Supplementary material for: Differential impact of divalent metals on native elongating transcript sequencing (NET-seq) protocols for RNA polymerases I and II
Source: PLoS One. 2025 Feb 13;20(2):e0315595. doi: 10.1371/journal.pone.0315595 (PMC11824990; doi:10.1371/journal.pone.0315595)
Supplement: S10 Table — (PDF) [file pone.0315595.s010.pdf]

	**1X**
**5X HF Phusion Buffer (ThermoScientific)**	16.7 μL
**10 mM each dNTPs (ThermoFisher, #R0181)**	1.7 μL
**Phusion Polymerase**	0.8 μL
**Sterile MilliQ Water**	58.4 μL
**Total Volume**	**77.6 μL**
