# Supplementary material for: Differential impact of divalent metals on native elongating transcript sequencing (NET-seq) protocols for RNA polymerases I and II
Source: PLoS One. 2025 Feb 13;20(2):e0315595. doi: 10.1371/journal.pone.0315595 (PMC11824990; doi:10.1371/journal.pone.0315595)
Supplement: S11 Table — (PDF) [file pone.0315595.s011.pdf]

Step	Temperature	Duration
1.	98°C	0:30
2.	98°C	0:10
3.	60°C	0:10
4.	72°C	1:00
5.	GOTO step 2	24X
6.	4°C	∞
