# Supplementary material for: Differential impact of divalent metals on native elongating transcript sequencing (NET-seq) protocols for RNA polymerases I and II
Source: PLoS One. 2025 Feb 13;20(2):e0315595. doi: 10.1371/journal.pone.0315595 (PMC11824990; doi:10.1371/journal.pone.0315595)
Supplement: S12 Table — (PDF) [file pone.0315595.s012.pdf]

	Sequence
**20 μM UMI Linker (IDT)**	5rApp/CANNNNNNNNCTCCACGAGTCATCCGC/3ddc/
**10 μM NET2 Primer (IDT)**	5Phos/CTGTAGGCACCATCAATGATCGTCGGA/isp18/CACTCA/isp18/CGTCTCTTCTGCGGATGACTCGTGGAG
