# Supplementary material for: Differential impact of divalent metals on native elongating transcript sequencing (NET-seq) protocols for RNA polymerases I and II
Source: PLoS One. 2025 Feb 13;20(2):e0315595. doi: 10.1371/journal.pone.0315595 (PMC11824990; doi:10.1371/journal.pone.0315595)
Supplement: S13 Table — (PDF) [file pone.0315595.s013.pdf]

Sample	Forward	Reverse
**Untreated 1**	CAAGCAGAAGACGGCATACGAGATttctgcctTCCGACGATCATTGATGGTGCC	AATGATACGGCGACCACCGAGATCTACACtagatcgcCGTCTCTTCTGCGGATGACTCG
**Untreated 2**	CAAGCAGAAGACGGCATACGAGATgctcaggaTCCGACGATCATTGATGGTGCC	AATGATACGGCGACCACCGAGATCTACACtagatcgcCGTCTCTTCTGCGGATGACTCG
**Untreated 3**	CAAGCAGAAGACGGCATACGAGATaggagtccTCCGACGATCATTGATGGTGCC	AATGATACGGCGACCACCGAGATCTACACtagatcgcCGTCTCTTCTGCGGATGACTCG
**$CaCl_2$ 1**	CAAGCAGAAGACGGCATACGAGATgctcaggaTCCGACGATCATTGATGGTGCC	AATGATACGGCGACCACCGAGATCTACACtagatcgcCGTCTCTTCTGCGGATGACTCG
**$CaCl_2$ 2**	CAAGCAGAAGACGGCATACGAGATaggagtccTCCGACGATCATTGATGGTGCC	AATGATACGGCGACCACCGAGATCTACACtagatcgcCGTCTCTTCTGCGGATGACTCG
**$CaCl_2$ 3**	CAAGCAGAAGACGGCATACGAGATcatgcctaTCCGACGATCATTGATGGTGCC	AATGATACGGCGACCACCGAGATCTACACtagatcgcCGTCTCTTCTGCGGATGACTCG
**$CaCl_2$ + MNase 1**	CAAGCAGAAGACGGCATACGAGATgtagagagTCCGACGATCATTGATGGTGCC	AATGATACGGCGACCACCGAGATCTACACtagatcgcCGTCTCTTCTGCGGATGACTCG
**$CaCl_2$ + MNase 2**	CAAGCAGAAGACGGCATACGAGATcctctctgTCCGACGATCATTGATGGTGCC	AATGATACGGCGACCACCGAGATCTACACtagatcgcCGTCTCTTCTGCGGATGACTCG
**$CaCl_2$ + MNase 3**	CAAGCAGAAGACGGCATACGAGATagcgtagcTCCGACGATCATTGATGGTGCC	AATGATACGGCGACCACCGAGATCTACACtagatcgcCGTCTCTTCTGCGGATGACTCG
**$MnCl_2$ 1**	CAAGCAGAAGACGGCATACGAGATgtagagagTCCGACGATCATTGATGGTGCC	AATGATACGGCGACCACCGAGATCTACACtagatcgcCGTCTCTTCTGCGGATGACTCG
**$MnCl_2$ 2**	CAAGCAGAAGACGGCATACGAGATcctctctgTCCGACGATCATTGATGGTGCC	AATGATACGGCGACCACCGAGATCTACACtagatcgcCGTCTCTTCTGCGGATGACTCG
**$MnCl_2$ 3**	CAAGCAGAAGACGGCATACGAGATagcgtagcTCCGACGATCATTGATGGTGCC	AATGATACGGCGACCACCGAGATCTACACtagatcgcCGTCTCTTCTGCGGATGACTCG
**$MnCl_2$ + DNase 1**	CAAGCAGAAGACGGCATACGAGATcagcctcgTCCGACGATCATTGATGGTGCC	AATGATACGGCGACCACCGAGATCTACACtagatcgcCGTCTCTTCTGCGGATGACTCG
**$MnCl_2$ + DNase 2**	CAAGCAGAAGACGGCATACGAGATtgcctcttTCCGACGATCATTGATGGTGCC	AATGATACGGCGACCACCGAGATCTACACtagatcgcCGTCTCTTCTGCGGATGACTCG
**$MnCl_2$ + DNase 3**	CAAGCAGAAGACGGCATACGAGATtcctctacTCCGACGATCATTGATGGTGCC	AATGATACGGCGACCACCGAGATCTACACtagatcgcCGTCTCTTCTGCGGATGACTCG
