# Supplementary material for: Differential impact of divalent metals on native elongating transcript sequencing (NET-seq) protocols for RNA polymerases I and II
Source: PLoS One. 2025 Feb 13;20(2):e0315595. doi: 10.1371/journal.pone.0315595 (PMC11824990; doi:10.1371/journal.pone.0315595)
Supplement: S14 Table — (PDF) [file pone.0315595.s014.pdf]

Software	Version
FastQC	0.11.7
fqtrim	0.9.7
cutadapt	3.4
Anaconda	5.3.1
STAR	2.7.1a
SAMTools	1.6
BEDTools	2.28.0
R	4.0.2
RStudio	1.3.959
dplyr	1.1.10
plyr	1.8.7
ggplot2	3.3.6
ggseqlogo	0.1
ggpubr	0.4.0
cowplot	1.1.1
matrixStats	0.61.0
hexbin	1.28.2
tweedie	2.3.5
statmod	1.4.36
magritter	2.0.3
scales	1.2.1
tidyr	1.2.1
zoo	1.8-11
DiffLogo	2.14.0
rclone	1.48.0
RcppRoll	0.3.0
