# Supplementary figures and images for: Differential impact of divalent metals on native elongating transcript sequencing (NET-seq) protocols for RNA polymerases I and II
Source: PLoS One. 2025 Feb 13;20(2):e0315595. doi: 10.1371/journal.pone.0315595 (PMC11824990; doi:10.1371/journal.pone.0315595)

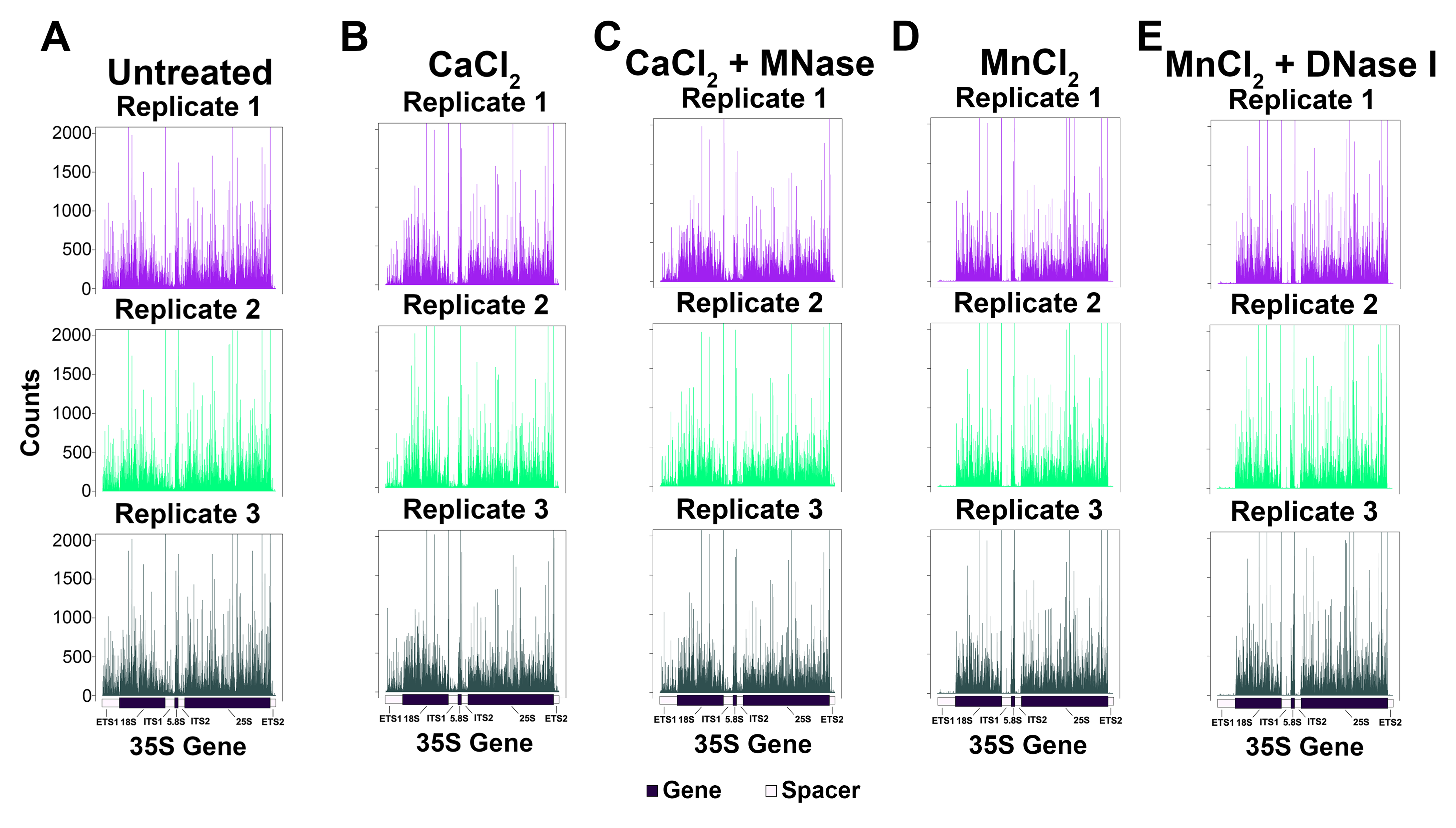

Supplement: S1 Fig — (TIF) [file pone.0315595.s015.tif]

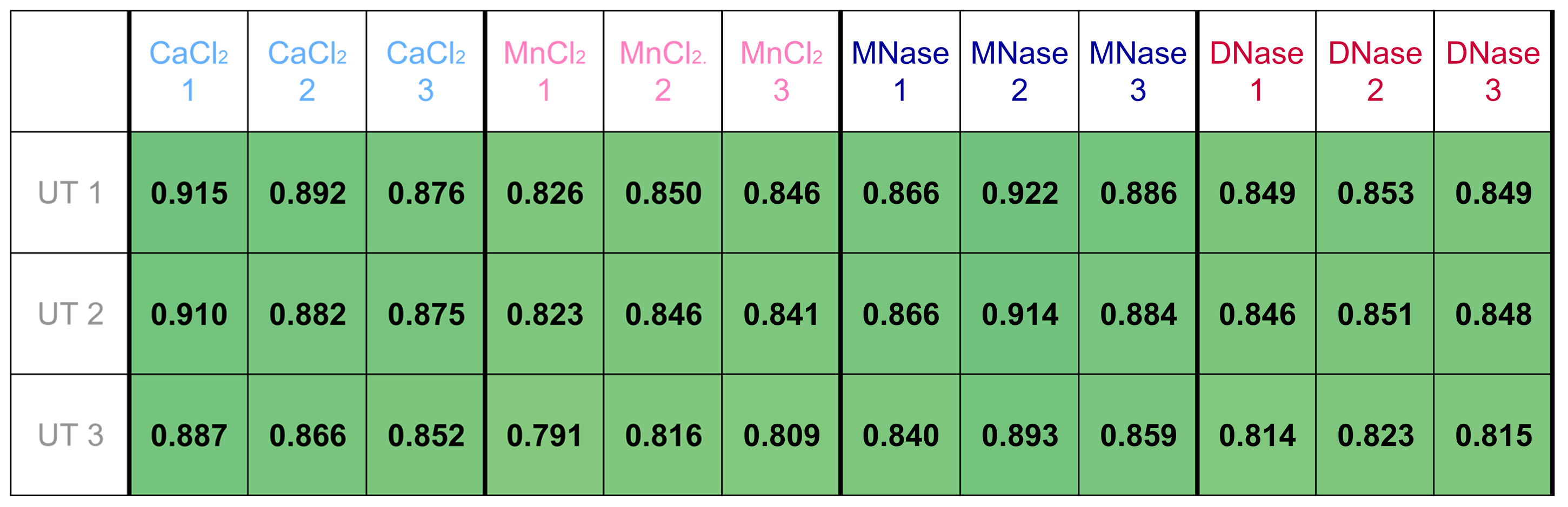

Supplement: S2 Fig — (TIF) [file pone.0315595.s016.tif]

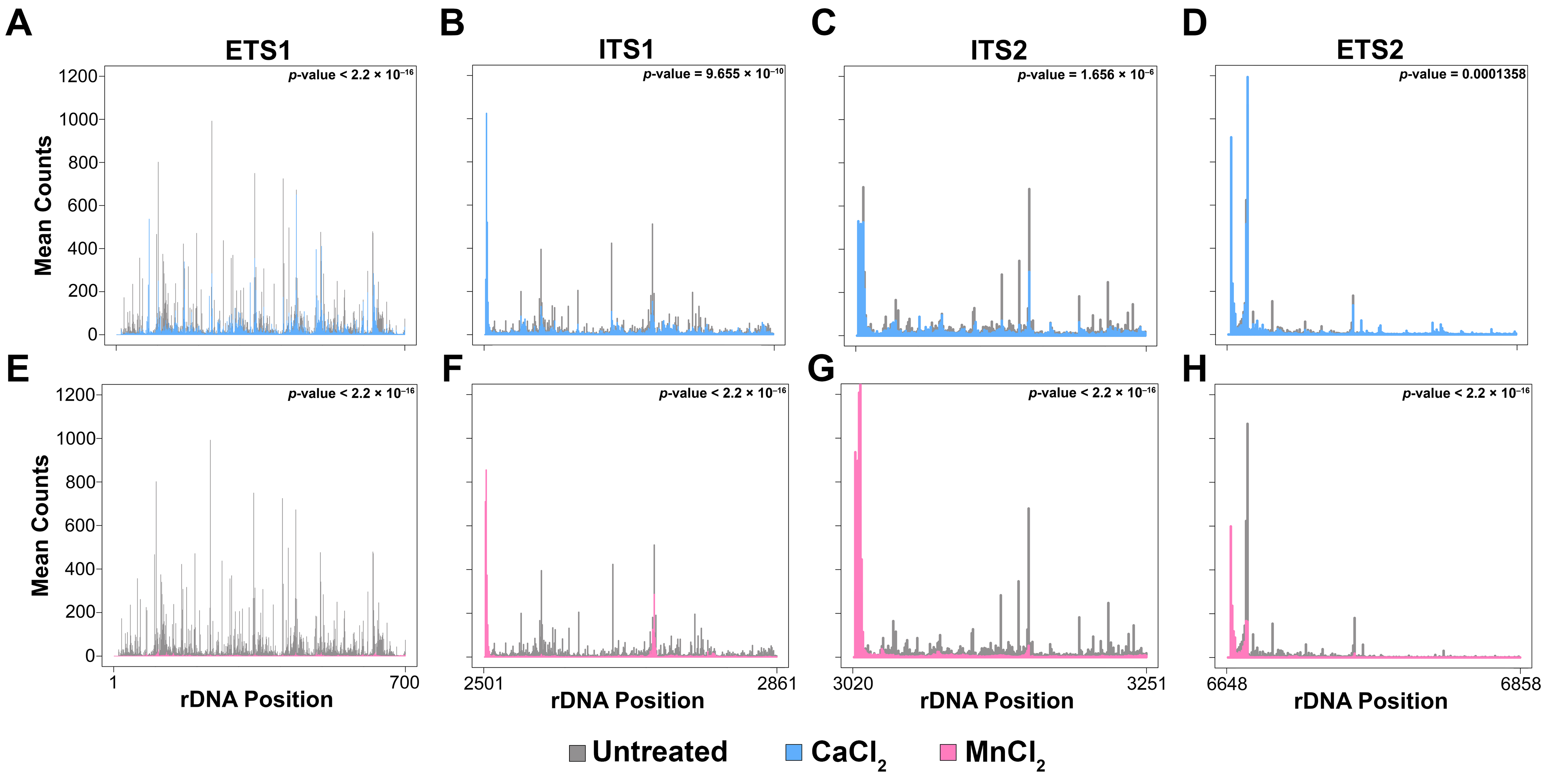

Supplement: S3 Fig — (TIF) [file pone.0315595.s017.tif]

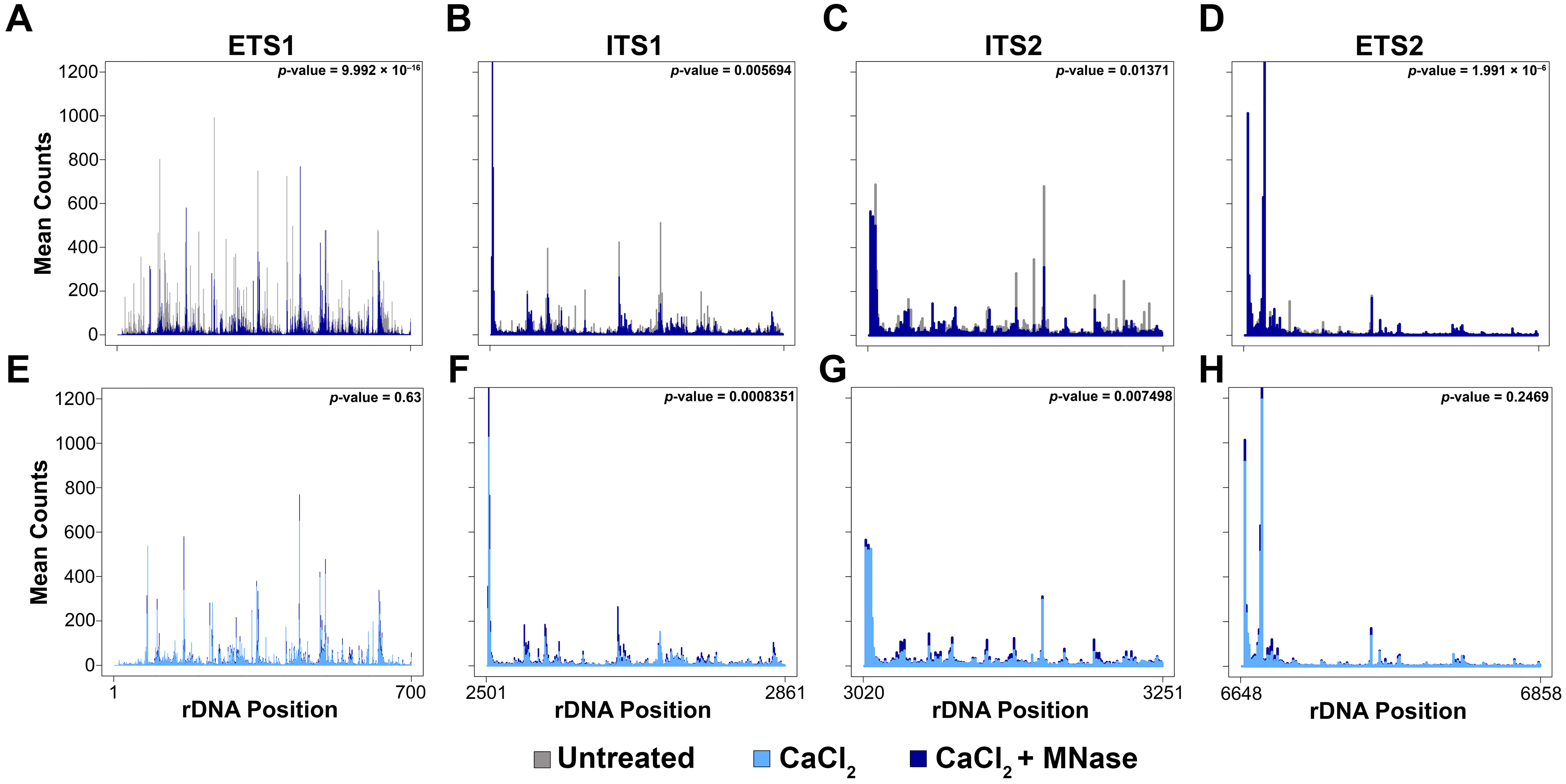

Supplement: S4 Fig — (TIF) [file pone.0315595.s018.tif]

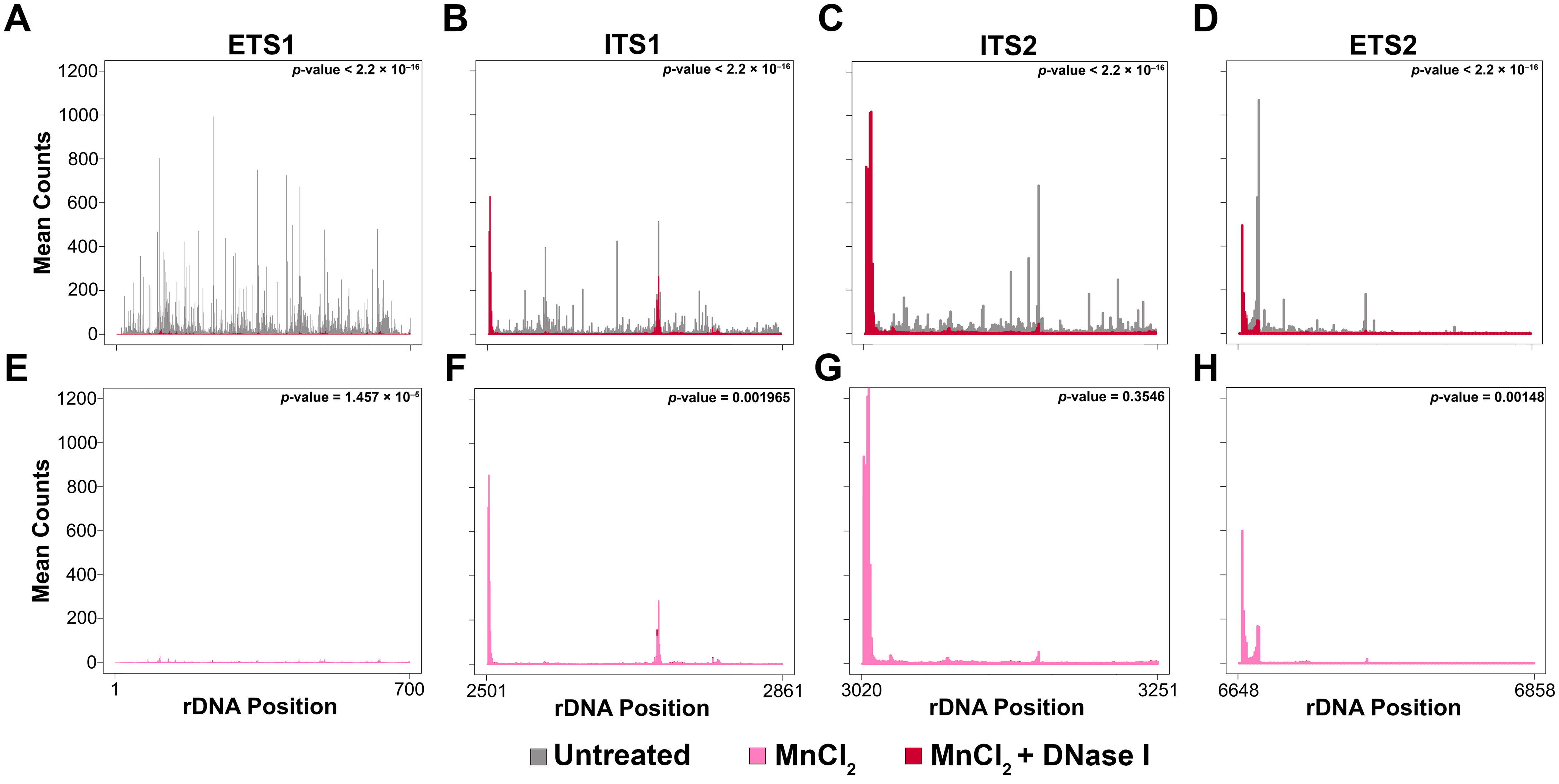

Supplement: S5 Fig — (TIF) [file pone.0315595.s019.tif]

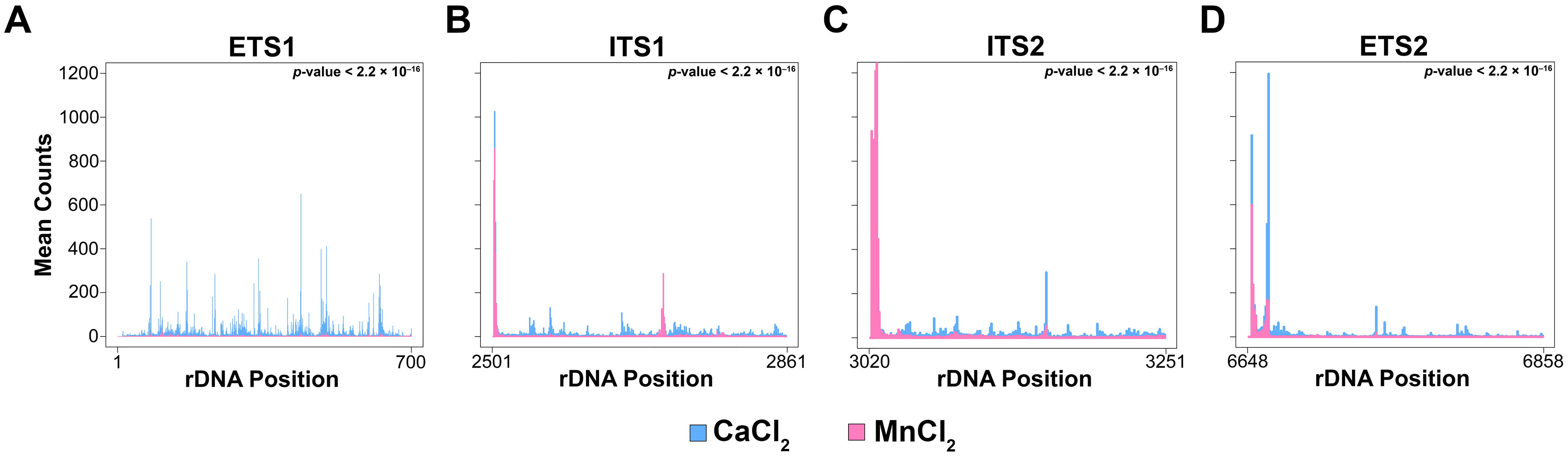

Supplement: S6 Fig — (TIF) [file pone.0315595.s020.tif]

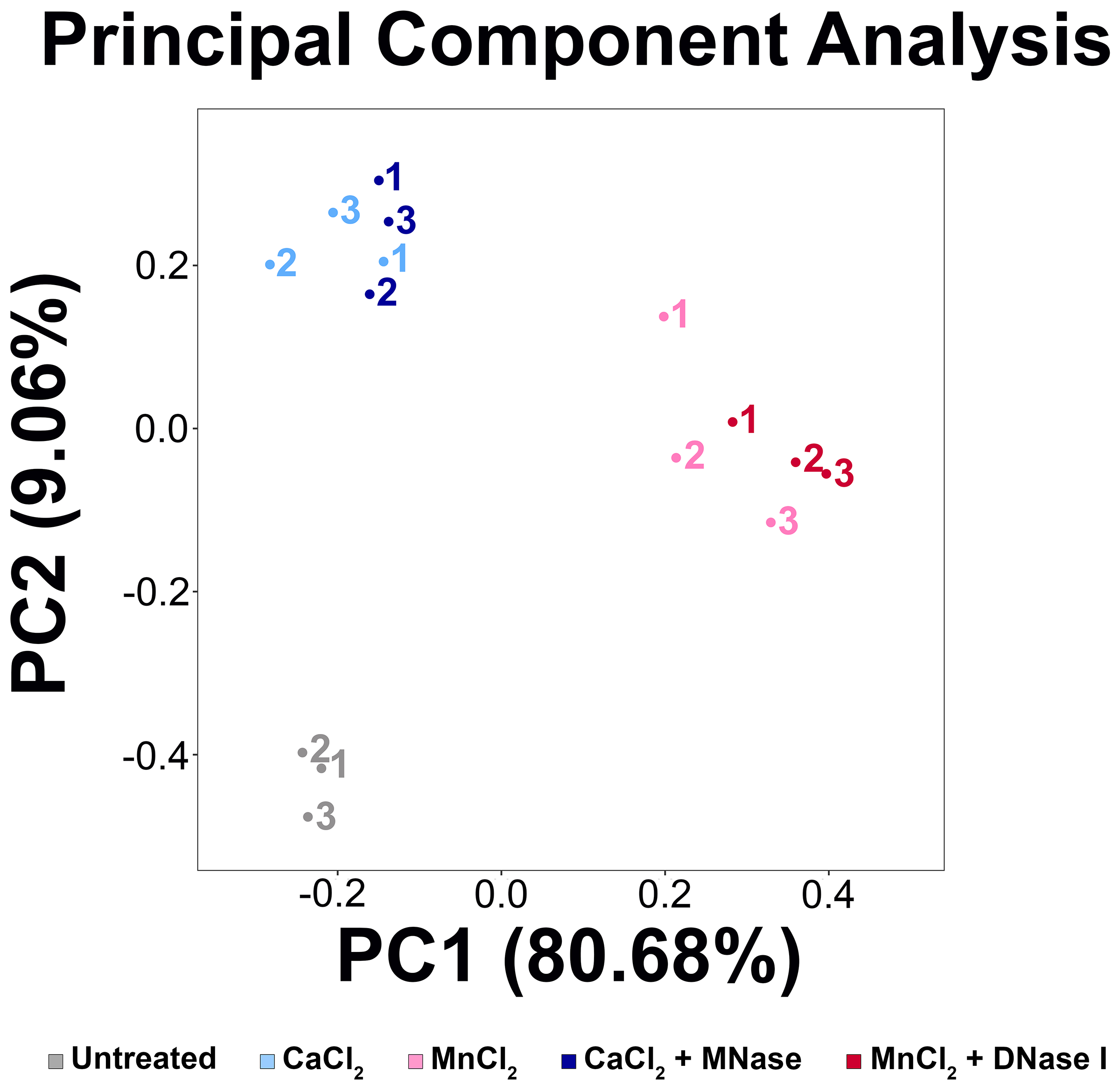

Supplement: S7 Fig — (TIF) [file pone.0315595.s021.tif]

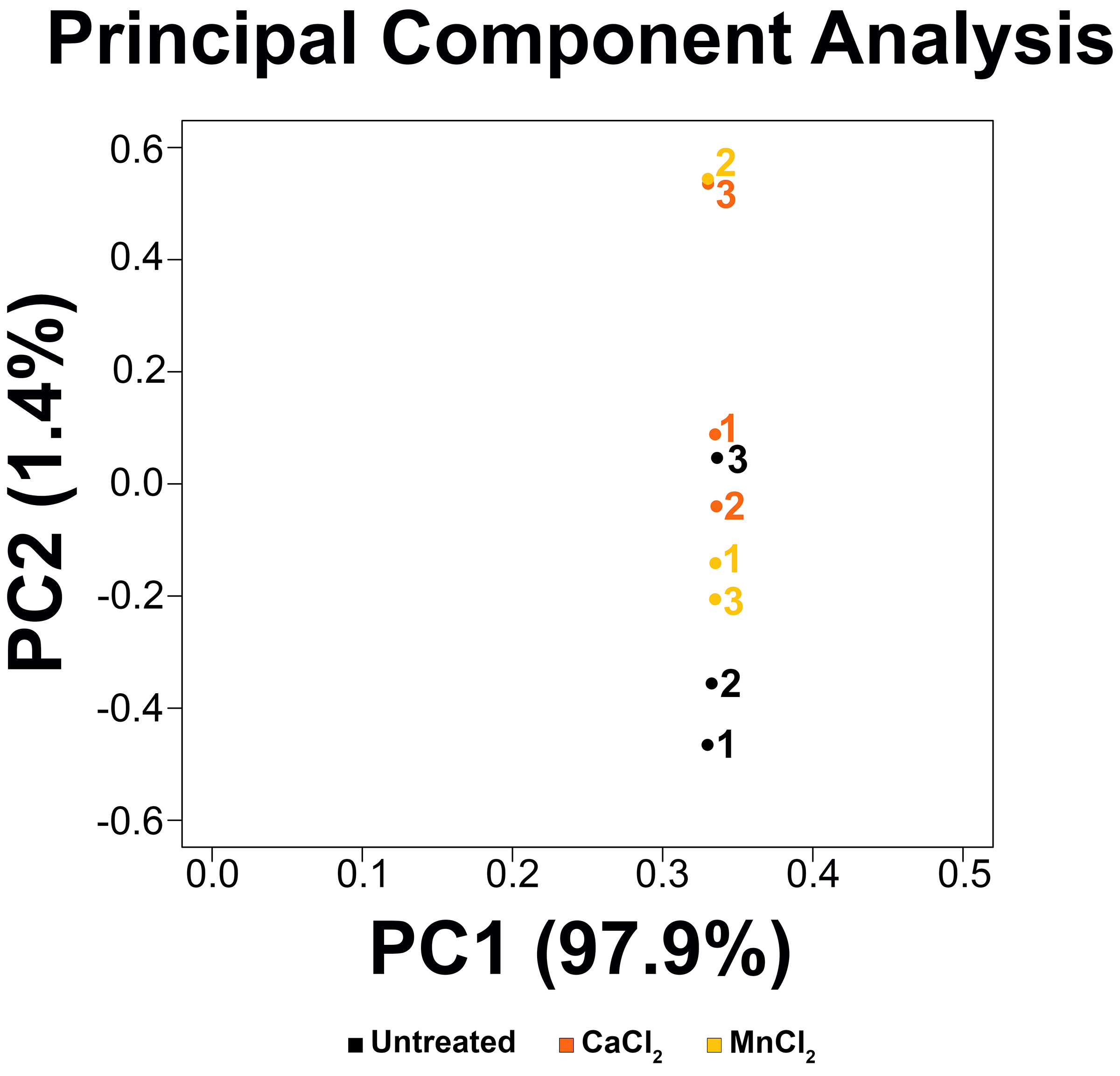

Supplement: S8 Fig — (TIF) [file pone.0315595.s022.tif]
