# Supplementary material for: Differential impact of divalent metals on native elongating transcript sequencing (NET-seq) protocols for RNA polymerases I and II
Source: PLoS One. 2025 Feb 13;20(2):e0315595. doi: 10.1371/journal.pone.0315595 (PMC11824990; doi:10.1371/journal.pone.0315595)
Supplement: S1 Text — (DOCX) [file pone.0315595.s023.docx]

**Extended NET-seq Protocol**

**Cell growth and harvest**

1. Streak and grow desired *Saccharomyces cerevisiae* (yeast) strain on a plate.
2. Inoculate a starter culture (25-40 mL) and grow overnight and grow to saturation at 30°C with nutation.
3. Using saturated starter culture, inoculate 1 liter of YEPD media and grow culture at 30°C with nutation until A_600_ = 0.3.
4. Meanwhile, set up glass collection apparatus by nesting a glass funnel with a fritted filter into a 1L Erlenmeyer flask with a side arm.
5. Once cells have reached A_600_ = 0.3, place a 0.45 μm Cellulose Nitrate Membrane Filter (GE Healthcare – Whatman, #7184-009) on top of the glass funnel and connect vacuum tubing to the flask. Turn the vacuum on.
6. Using a clip, secure a glass collection cup on top of the glass funnel.
7. Quickly pour the entire 1L culture into the collection cup and allow all media to filter into the bottom flask, leaving behind a layer of yeast cells on top of the filter.
8. Using a flamed and freshly sterilized metal spatula, quickly scrape the yeast cells off the filter.
9. Submerge the spatula with the scraped cells into a conical containing liquid nitrogen.
10. Flash freeze cells for approximately 1-2 minutes and use another flamed and freshly sterilized metal spatula to scrape the cells off the first spatula and into the conical.
11. Screw the cap onto the conical, but do not tighten.
12. Carefully invert conical to remove excess liquid nitrogen.
13. Store frozen cells at -80°C until ready for use (these cells can be stored for many weeks or months in the freezer as long as they have not been lysed).

**Cell lysis (with Mikro-Dismembrator II)**

1. Collect dry ice into a cooler.
2. Dispense liquid nitrogen into a small cryogenic bowl with a wire cage nested inside.
3. Place a clean and fully dried plastic grinding cup containing a metal ball inside the wire cage and fully submerge the grinding cup in the liquid nitrogen.
4. After approximately 3-5 minutes, transfer the entire yeast sample immediately from the freezer into the cold grinding cup.
5. Submerge the grinding cup in the liquid nitrogen for 1 minute.
6. Using a Mikro-Dismembrator II, lyse cells at maximum speed (16 mm) for 1 minute.
7. Once time has elapsed, remove the grinding cup from the machine and submerge in liquid nitrogen for 1 minute.
8. Repeat steps 6 and 7 for a total of 10 lysis/rest cycles.
9. After lysis is complete, remove the grinding cup from the liquid nitrogen and use a flamed and freshly sterilized metal spatula to scrape the lysed cells from the cup into a conical placed on dry ice (note – record the weight of the grindate by weighing the conical before and after scraping the cells into it).
10. Store lysed cells at -80°C (grindates should be used within 24-48 hours of lysis).

**Cell lysis (with CoolTeenPrep adapter)**

1. Collect dry ice into a cooler.
2. Label, weigh, and record weights of empty TeenPrep Lysing Matrix A (15 mL) conicals and place on dry ice.
3. Transfer frozen yeast cell pellet into labelled TeenPrep conicals and return to dry ice.
4. Load the CoolTeenPrep adapter with dry ice (according to manufacturer’s instructions).
5. Place sample tubes (make sure to balance the tubes inside the adapter similar to loading a centrifuge) and secure adapter in the FastPrep.
6. Lyse cells for 1 minute.
7. Remove tubes from adapter and let them rest on dry ice for one minute.
8. Repeat steps 6 and 7 for a total of 5 lysis/rest cycles.
9. After lysis is complete, weigh tubes containing samples and record weights.
10. Label 50 mL conicals (one per sample) and dump as much of sample/bead mixture into conical (this step is optional, but it can help to avoid sample loss during resuspension with lysis buffer during step 3 of the “Immunoprecipitation and RNA Extraction” section).
11. Store lysed cells (either just the 15 mL conical or the 15 mL + 50 mL conicals if including step 10) at -80°C (grindates should be used within 24-48 hours of lysis).

**Blocking beads for immunoprecipitation**

1. One day prior to following the protocol in the “Immunoprecipitation and RNA extraction” section, block Pierce Anti-HA magnetic beads (ThermoFisher Scientific, #88836) overnight using the steps below.
2. Prepare the appropriate amount of 1X lysis buffer (4 mL per sample; 10X lysis buffer recipe in S1 Table and 1X lysis buffer recipe in S2 Table) and 1X blocking buffer (0.5 mL per sample; S3 Table) needed based on the number of samples. Add water first, then the rest of the reagents in the order listed in the tables below.

**S1 Table. 10X lysis buffer (can be prepared ahead of time and stored at room temperature).**

**S2 Table. 1X lysis buffer with EDTA included (freshly prepared for each experiment).**

**S3 Table. 1X blocking buffer (freshly prepared for each experiment).**

1. Mix the lysis and blocking buffers vigorously and then place on ice.
2. Add the appropriate amount of Pierce Anti-HA magnetic beads ((grindate weight calculated after lysing cells * 5) * (0.04)) to a low-retention tube for each sample.
3. Place tubes onto a magnetic tube rack to collect the beads on the side of the tubes for approximately 30 seconds.
4. Remove the buffer from each tube, quickly resuspend the beads in each tube in 1 mL of 1X lysis buffer, and place tubes on ice.
5. Wash beads at 4°C for 2 minutes with inversion on a tube rotator.
6. Repeat steps 5-7 for a total of 3 washes.
7. After completing 3 washes, place the tubes onto a magnetic tube rack for approximately 30 seconds.
8. Remove the buffer from each tube, then resuspend beads in each tube in 0.5 mL of 1X blocking buffer.
9. Incubate the tubes with the beads overnight at 4°C with inversion on a tube rotator.

**Immunoprecipitation and RNA Extraction**

1. Prepare the appropriate amount of 1X lysis buffer with EDTA (5 mL * grindate weight for each sample; S2 Table) for the untreated samples, 1X lysis buffer without EDTA (5 mL * grindate weight for each sample; S4 Table) for CaCl_2_, CaCl_2_ + MNase, MnCl_2_, and MnCl_2_ + DNase I samples, and 1X wash buffer (4 mL per sample; S5 Table). Add water first, then add the rest of the reagents in the order they are listed in the tables.

Mix lysis and wash buffers well and place on ice.

**S4 Table. 1X lysis buffer without EDTA (freshly prepared for each experiment).**

**S5 Table. 1X wash buffer (freshly prepared for each experiment)**

1. Quickly dissolve grindate in appropriate amount of 1X lysis buffer (with EDTA for the untreated samples and without EDTA for the 4 sets of samples subjected to treatment; 5 mL * grindate weight calculated after lysis) while samples are on ice.
2. Use a glass pipet to stir the grindate and pipet up and down to thoroughly dissolve cells in lysis buffer.
3. Repeat steps 2 and 3 to completely resuspend all samples in 1X lysis buffer while incubating on ice.
4. For the untreated samples, proceed directly to step 6. For CaCl_2_ and CaCl_2_ + MNase treated samples, proceed to “CaCl_2_ and CaCl_2_ + MNase Samples” section below. For MnCl_2_ and MnCl_2_ + DNase I treated samples, proceed to “MnCl_2_ and MnCl_2_ + DNase I Samples” section below.

*CaCl_2_ and CaCl_2_ + MNase Samples:*

1. With samples on ice, add CaCl_2_ at a final concentration of 5 mM to both the CaCl_2_ and CaCl_2_ + MNase treated samples and mix well.
2. Add MNase (Worthington, #LS004797) at a final concentration of 100 U/μL to CaCl_2_ + MNase treated samples only and mix well.
3. Incubate both CaCl_2_ and CaCl_2_ + MNase treated samples at 37°C for 2 minutes.
4. Add EDTA (pH 8.5) at a final concentration of 5 mM to both the CaCl_2_ and CaCl_2_ + MNase treated samples and mix well. Place samples on ice.
5. Proceed to step 6.

*MnCl_2_ and MnCl_2_ + DNase I Samples:*

1. With samples on ice, add MnCl_2_ at a final concentration of 10 mM to both the MnCl_2_ and MnCl_2_ + DNase I treated samples and mix well.
2. Add DNase I (NEB, #M0303L) at a final concentration of 100 U/mL to the MnCl_2_ + DNase I treated samples only and mix well.
3. Incubate both the MnCl_2_ and MnCl_2_ + DNase I treated samples on ice for 20 minutes.
4. Add EDTA (pH 8.5) at a final concentration of 5 mM to both the MnCl_2_ and MnCl_2_ + DNase I treated samples and mix well. Place samples on ice.
5. Incubate both the MnCl_2_ and MnCl_2_ + DNase I treated samples on ice for 30 minutes.
6. Proceed to step 6.
7. Aliquot each sample into pre-chilled low retention tubes and spin samples at 16,873 x g for 15 minutes at 4°C.
8. Collect the supernatant for each sample into 1 conical per sample and place on ice.
9. Place tubes containing the Anti-HA magnetic beads and blocking buffer (blocked overnight at 4°C) onto the magnetic tube rack and let beads settle to the side of each tube for approximately 30 seconds.
10. Remove the blocking buffer from each tube.
11. Resuspend the beads for each sample in 1 mL of sample lysate and transfer the beads + 1 mL lysate back to the sample conical (beads and lysate will now be in the same conical).
12. Repeat step 10 for each sample until the beads have been incorporated into each sample conical.
13. Incubate samples for 3 hours at 4°C with inversion on a tube rotator.
14. After three hours, remove the tubes and place on ice.
15. Aliquot each sample into pre-chilled low retention tubes.
16. Place tubes on the magnetic tube rack, allowing beads to settle to the side of each tube for approximately 30 seconds.
17. Discard the supernatant from each tube and resuspend beads in 1 mL of 1X wash buffer.
18. Wash beads for each sample for 2 minutes at 4°C with inversion on a tube rotator.
19. Repeat steps 15-17 for a total of four washes.
20. After the final wash, place the tubes on the magnetic tube rack and allow beads to settle to the side of each tube for approximately 30 seconds.
21. Discard the wash buffer from each tube and resuspend the beads for each sample in 500 μL of TES (10 mM Tris-HCl (pH 7.5), 1 mM EDTA (pH 8.5), 1% SDS).
22. Add 500 μL of phenol ((pH 4.3) Fisher, #BP1751I-400) to each tube and vortex to mix.
23. Centrifuge samples for 5 minutes at 17,000 x g at room temperature.
24. Transfer the aqueous layer for each sample to a new tube.
25. Repeat steps 21-23 until completing 2 total phenol extractions and 2 addition extractions with chloroform (Thermo, #AA32614K2).
26. After the final chloroform extraction, transfer the aqueous layer into a new tube.
27. Prepare a glycoblue solution in a new tube with a 1:5 ratio of GlycoBlue Coprecipitant (Invitrogen, #AM9515) to sterile MilliQ water.
28. Add 10 μL of the glycoblue solution (from step 27) to each sample.
29. Add 1.4 mL ammonium acetate precipitation solution (1 M ammonium acetate, 95% ethanol) to each sample.
30. Precipitate samples at -80°C overnight or longer (can precipitate samples for weeks or even months).

**Linker ligation, enzymatic linker digestion, and zinc fragmentation**

1. Centrifuge sample tubes at 16,873 x g for 1 hour at 4°C.
2. After spin, remove tubes and discard all of the precipitation solution (taking care not to disturb the RNA pellet at the bottom of the tube).
3. Add 750 μL of 75% ethanol to each tube.
4. Centrifuge samples at 17,000 x g for 2 minutes at room temperature.
5. Repeat steps 2-4 until two total ethanol washes have been completed.
6. After the final wash, carefully remove the ethanol and leave the caps of the tubes open until all remaining residual ethanol has evaporated.
7. Dissolve RNA pellets in 11.5 μL of 10 mM Tris-HCl (pH 6.9) - only need 10 μL of sample for linker ligation reaction but will use ~1.5 μL of sample to check the RNA concentration.
8. Using a nanodrop, determine the RNA concentration and quality of each sample.
9. Make the ligation mix without the linker (will add this after denaturing) by adding the reagents in the order they are listed in the table (S6 Table), scaling up based on the number of samples. Place the mix on ice. The sequence of the UMI linker is included in S12 Table.

**S6 Table. Ligation mix for a single sample.**

1. Mix ligation mix vigorously.
2. Add the appropriate amount of the UMI linker to a PCR tube.
3. Denature the UMI linker for 3 minutes at 80°C, then immediately place on ice.
4. After denaturing, add the entire amount of the denatured UMI linker to the ligation mix and thoroughly mix.
5. Transfer each RNA sample (10 μL) to a new PCR tube.
6. Denature the RNA samples for 2 minutes at 80°C, then immediately place the tubes on ice or onto a chilled tube rack.
7. Add 9 μL of the ligation mix to each sample and mix well.
8. Add 1 μL of T4 RNA Ligase 2, truncated (NEB, #M0242L) to each sample and mix well.
9. Incubate samples at 25°C for 3 hours.
10. After 3 hours have elapsed, add 2 μL of 5’ Deadenylase (NEB, #M0331S) to each sample and mix well.
11. Incubate samples at 30°C for 45 minutes.
12. Dilute samples 2.5X with sterile MilliQ water supplemented 0.6X with NEBuffer 2 (NEB, #B7002S). Therefore, for a sample volume of 22 μL in volume, add 19.8 μL of NEBuffer 2 and 13.2 μL of sterile MilliQ water. Mix well.
13. Add 2 μL of RecJ_f_ (NEB, #M0264L) to each sample and mix well.
14. Incubate samples at 37°C for 45 minutes.
15. Add 2.2 μL of fragmentation solution (100 mM Tris-HCl (pH 6.9), 100 mM ZnCl_2_) to each sample and mix well.
16. Incubate samples at 70°C for 16 minutes.
17. After fragmentation is complete, immediately place tubes on ice or onto a chilled tube rack.
18. Add 2.5 μL of 200 mM EDTA (pH 8.5) to each sample and mix well.
19. Transfer samples to a new tube, then add 1 μL glycoblue and 360 μL of ammonium acetate precipitation solution (see recipe in “Immunoprecipitation and RNA Extraction” section) to each sample.
20. Precipitate samples at -80°C overnight or longer (can precipitate samples for weeks or even months).

**Reverse transcription and size selection**

1. Centrifuge sample tubes at 16,873 x g for 1 hour at 4°C.
2. After spinning, discard the remaining ammonium acetate precipitation solution from each sample tube (being careful not to disturb the pellet at the bottom of the tube).
3. Add 750 μL of 75% ethanol to each sample, then centrifuge samples at 17,000 x g for 2 minutes at room temperature.
4. Repeat steps 2 and 3 until a total of 2 ethanol washes have been completed.
5. After removing the ethanol from the second wash, leave the caps of the tubes open to allow for the residual ethanol to completely evaporate from the pellet.
6. Dissolve pellets in 10 μL of 10 mM Tris-HCl (pH 6.9).
7. Prepare the reverse transcription and the RNasin/DTT mixes by adding reagents in the order that they are listed in the tables (S7 and S8 Tables), scaling up based on the number of samples. Place the mixes on ice. The oligo sequence for the NET2 primer is included in S12 Table.

**S7 Table. Reverse transcription mix for a single sample.**

**S8 Table. RNasin/DTT mix for a single sample.**

1. Mix the reverse transcription mix vigorously, then transfer 5.4 μL to one PCR tube per sample.
2. Add the entire 10 μL of each sample to PCR tubes (one sample per tube) and mix well to incorporate samples with the reverse transcription mix.
3. Incubate samples at 65°C for 5 minutes.
4. Immediately place the tubes on ice or onto a chilled tube rack.
5. Mix the RNasin/DTT mix vigorously, then add 1.32 μL to each sample and mix well.
6. Add 0.82 μL of Superscript III Reverse Transcriptase (Invitrogen, #56575) to each sample and mix well.
7. Incubate samples at 45°C for 30 minutes.
8. Add 1.8 μL of 1 M NaOH to each sample and mix well.
9. Incubate samples at 98°C for 20 minutes.
10. After reverse transcription is complete, combine samples with 20 μL of loading dye (90% formamide, 25 μM EDTA (pH 8.5), 0.025 μg/μL Bromophenol Blue).
11. Heat samples at 100°C for 5 minutes.
12. Load samples (leaving an entire well blank between samples or loading only loading dye into the wells between samples) onto a 10% polyacrylamide gel and run the gel at 700V for approximately 1 hour.
13. Stain the gel with 100 mL of staining solution (SYBR Gold (Invitrogen, S11494) diluted 1:10,000 with 1X TBE) at room temperature for 20 minutes with rotation.
14. Meanwhile, use a sterilized 20G needle to poke a hole in the bottom of a 0.5 mL tube and nest the tube inside of a 1.5 mL tube (prepare one nested tube inside a larger tube per sample).
15. After staining, image gel.
16. Use sterile razorblades to excise samples from the gel between 120 bp (large primer band will be located just below this) and 600 bp.
17. Once a sample has been excised from the gel, place it into the appropriately labelled nested tube. Repeat steps 23 and 24 for all samples.
18. Centrifuge the samples at 17,000 x g for 3 minutes at room temperature.
19. Transfer any gel pieces that remain in the nested tube into the bottom tube after the spin for each sample.
20. Add 500 μL of sterile MilliQ water to each sample.
21. Incubate samples at -80°C for 15 minutes.
22. Incubate samples at 70°C for 15 minutes.
23. Incubate samples at 30°C with rotation overnight.
24. The next day, use a mini centrifuge to collect the gel slurry at the bottom of the tubes.
25. Transfer the gel slurry for each sample into a Costar Spin-X centrifuge tube filter (Corning, #8160).
26. Centrifuge samples at 17,000 x g for 3 minutes at room temperature.
27. Discard the gel pieces collected in the top filter for each sample.
28. Transfer the liquid sample from the bottom of the tube into a new tube for each sample.
29. Add 32 μL of 3 M NaCl to each sample.
30. Add 940 μL of 100% isopropanol to each sample.
31. Add 1 μL of glycoblue to each sample.
32. Precipitate samples at -80°C overnight or longer (can precipitate samples for weeks or even months).

**Circularization and library amplification/preparation for sequencing**

1. Centrifuge sample tubes at 16,873 x g for 1 hour at 4°C.
2. Remove and discard the ammonium acetate precipitation solution from all samples (being careful not to disturb the pellet at the bottom of the tube).
3. Add 750 μL of 75% ethanol and centrifuge samples at 17,000 x g for 2 minutes at room temperature.
4. Repeat steps 2 and 3 until pellets have been washed with 75% ethanol for a total of 2 times.
5. After the final wash, remove the ethanol and leave the caps of the tubes open to allow for all residual ethanol to evaporate.
6. Dissolve the pellets in 15 μL of 10 mM Tris-OAc (pH 7.9).
7. Prepare the circularization mix by adding the reagents in the order they are listed in the table (S9 Table), scaling up based on the number of samples. Mix well, then place mix on ice.

**S9 Table. Circularization mix for a single sample.**

1. Add 4 μL of the circularization mix to one PCR tube per sample.
2. Add the entire sample (15 μL) to each PCR tube (one sample per tube) and mix well.
3. Add 1 μL of CircLigase ssDNA Ligase (Biosearch Technologies, #CL4111K) to each sample and mix well.
4. Incubate samples at 60°C for 1 hour.
5. Add an additional 1 μL of CircLigase ssDNA Ligase to each sample and mix well.
6. Incubate samples at 60°C for 1 hour.
7. Incubate samples at 80°C for 10 minutes.
8. Transfer circularized DNA to a new tube for storage (circDNAs are very stable and excess sample can be stored at -20°C long-term).
9. Prepare the phusion master mix by adding the reagents in the order they appear in the table (S10 Table), scaling up based on the number of samples. Mix well and place mix on ice. The Phusion polymerase was previously purified by our lab in-house.

**S10 Table. Phusion master mix for a single sample.**

1. For each sample, make a unique library amplification mix by combining 77.6 μL of phusion master mix + 0.8 μL forward primer + 0.8 μL reverse primer (see S13 Table for forward and reverse primer sequences).
2. Add 1 μL of circularized DNA of each sample to a PCR tube.
3. To each sample, add 16.7 μL of the unique library amplification mix and mix well.
4. Use a thermal cycler to amplify libraries based on the cycle provided in S11 Table (25 amplification cycles total). Set lid temperature to 105°C.

**S11 Table. Library amplification cycle protocol.**

1. After amplification, use PCRClean DX Beads (Aline, #C-1003-5) to prepare samples for sequencing by following the manufacturer’s protocol exactly.

**S12 Table. Oligo sequences for the UMI linker and reverse transcription primer.**

**S13 Table. Oligo sequences for library amplification indices for each sample.**

**S14 Table. R packages and versions used in data analysis.**

**S1 Figure. Histograms for individual replicates display reproducibility within treatment conditions.** Histograms were plotted demonstrating individual occupancy patterns for the three replicates within each treatment group. Each panel (A-E) displays the data for a different treatment condition.

**S2 Figure. Spearman correlation coefficient values for untreated vs. treated samples.** The Spearman correlation test was performed for each of the untreated (UT) samples against every single one of the treated samples.

**S3 Figure. Divalent cation treatment reduces Pol I occupancy across the spacer regions of the rDNA.** The mean counts for the untreated samples were overlaid with the mean counts for either the CaCl_2_ treated samples (A-D) or the MnCl_2_ treated samples (E-H) in each of the four spacer regions of the rDNA. The Kolmogorov-Smirnov test was performed to assess whether there was a significant difference in the distribution patterns between treatment groups, and the resulting *p*-value is inset in the upper righthand corner of each panel.

**S4 Figure. CaCl_2_ treatment causes a significant reduction in Pol I occupancy, but the addition of MNase only results in mild changes to occupancy in the spacer regions.** (A-D) The mean counts for the untreated samples were overlaid with the mean counts for the CaCl_2_ + MNase treated samples in each of the four spacer regions of the rDNA. (E-H). The mean counts for the CaCl_2_ treated samples were overlaid with the mean counts for the CaCl_2_ + MNase treated samples in each of the four spacer regions of the rDNA. Resulting *p*-values from the Kolmogorov-Smirnov test are included in the upper righthand corner of each panel.

**S5 Figure. MnCl_2_ treatment causes a significant reduction in Pol I occupancy and DNase I does not have an additional effect in the spacer regions.** (A-D) The mean counts for the untreated samples were overlaid with the mean counts for the MnCl_2_ + DNase I treated samples in each of the four spacer regions of the rDNA. (E-H). The mean counts for the MnCl_2_ treated samples were overlaid with the mean counts for the MnCl_2_ + DNase I treated samples in each of the four spacer regions of the rDNA. Resulting *p*-values from the Kolmogorov-Smirnov test are included in the upper righthand corner of each panel.

**S6 Figure. MnCl_2_ treatment causes a more severe reduction in Pol I occupancy as compared to CaCl_2_ treatment in the spacer regions.** (A-D) The mean counts for the CaCl_2_ treated samples were overlaid with the mean counts for the MnCl_2_ samples in each of the four spacer regions of the rDNA. Resulting *p*-values from the Kolmogorov-Smirnov test are included in the upper righthand corner of each panel.

**S7 Figure. Principal component analysis (PCA) plot for Pol I samples.** A PCA was performed to investigate clustering patterns between replicates within treatment conditions for Pol I samples. Replicates are labelled in the plot.

**S8 Figure. PCA plot for Pol II samples.** Using deepTools, a PCA was performed to investigate clustering patterns between replicates within treatment conditions for Pol II samples. Replicates are labelled in the plot.
